# Structural insights into the specific recognition of mitochondrial ribosome-binding factor hsRBFA and 12 S rRNA by methyltransferase METTL15

**DOI:** 10.1038/s41421-023-00634-z

**Published:** 2024-01-30

**Authors:** Mengqi Lv, Wanwan Zhou, Yijie Hao, Fudong Li, Huafeng Zhang, Xuebiao Yao, Yunyu Shi, Liang Zhang

**Affiliations:** 1 Hefei National Research Center for Cross Disciplinary Science, School of Life Sciences, Division of Life Sciences and Medicine, University of Science and Technology of China, Hefei, Anhui China; 2 Ministry of Education Key Laboratory for Membraneless Organelles and Cellular Dynamics, University of Science & Technology of China, Hefei, Anhui China; 3 Center for Advanced Interdisciplinary Science and Biomedicine of IHM, Division of Life Sciences and Medicine, University of Science and Technology of China, Hefei, Anhui China

**Keywords:** Post-translational modifications, Nanocrystallography

## Abstract

Mitochondrial rRNA modifications are essential for mitoribosome assembly and its proper function. The m^4^C methyltransferase METTL15 maintains mitochondrial homeostasis by catalyzing m^4^C839 located in 12 S rRNA helix 44 (h44). This modification is essential to fine-tuning the ribosomal decoding center and increasing decoding fidelity according to studies of a conserved site in *Escherichia coli*. Here, we reported a series of crystal structures of human METTL15–hsRBFA–h44–SAM analog, METTL15–hsRBFA–SAM, METTL15–SAM and apo METTL15. The structures presented specific interactions of METTL15 with different substrates and revealed that hsRBFA recruits METTL15 to mitochondrial small subunit for further modification instead of 12 S rRNA. Finally, we found that METTL15 deficiency caused increased reactive oxygen species, decreased membrane potential and altered cellular metabolic state. Knocking down METTL15 caused an elevated lactate secretion and increased levels of histone H4K12-lactylation and H3K9-lactylation. METTL15 might be a suitable model to study the regulation between mitochondrial metabolism and histone lactylation.

## Introduction

As a major source of ATP, mitochondria are essential for the process of cellular energy production through oxidative phosphorylation (OXPHOS). Mitochondrial dysfunction causes defects in OXPHOS activity, disorder of cell energy metabolism, increases in reactive oxygen species (ROS), oxidative stress and cell death, which are closely related to aging, diabetes, neurodegenerative diseases, cardiovascular diseases and cancers^1–6^.

Mammalian mitochondria retain a unique ~16.5 kb circular DNA genome, which encodes 2 mitochondrial ribosomal RNAs (12 S and 16 S), 22 mitochondrially-encoded transfer RNAs and 13 protein components of the OXPHOS system. Mammalian mitochondrial ribosomes (mitoribosomes), which are responsible for the translation of the 13 protein component genes, are composed of the 28 S mitochondrial small subunit (mt-SSU, containing 12 S rRNA and 30 nucleus-encoded mitoribosomal proteins) and 39 S mitochondrial large subunit (mt-LSU, containing 16 S rRNA and 52 nucleus-encoded mitoribosomal proteins)^7–9^. A series of post-transcription modifications have been found in mitochondrial rRNAs, which are located at the functionally critical regions of the mitoribosome and play a crucial role in mitoribosome assembly and efficient translation^10–12^. Abnormal expression of mt-rRNA modification enzymes directly affects modification levels of mt-rRNA and mitoribosome assembly, which damages mitochondria function and leads to a series of mitochondrial diseases^13,14^. For example, methyltransferase TFB1M, a potential risk gene for type 2 diabetes in humans, dimethylates 12 S rRNA A936 and A937 and regulates the assembly of mitoribosome^15^. *Tfb1m* homozygous knockout (KO) mice were embryonically lethal, while mitochondrial damage and decreased insulin secretion in response to glucose occurred in the islets of mice heterozygous for *Tfb1m* deficiency^16,17^.

Methyltransferase-like (METTL) family proteins are characterized by a conserved Rossman-like fold S-adenosyl methionine (SAM)-binding domain, which methylates proteins, nucleic acids, and other small molecule metabolites, are involved in the regulation of mRNA stability and translation efficiency^18–20^. Several METTLs localize to mitochondria, among which METTL9, METTL12 and METTL20 are responsible for protein methylation, while METTL8 and METTL2A for mt-tRNA methylation, METTL15 for mt-rRNA methylation^10,21–23^.

The N4-methylcytidine (m^4^C) methyltransferase METTL15 has been identified as responsible for specific recognition and modification of m^4^C839 in the 44th cervical ring structure of mt-SSU 12 S rRNA (helix 44)^24–26^. The 12 S mt-rRNA m^4^C839 modification is highly conserved to that in *Escherichia coli* SSU 16 S rRNA m^4^C1402, which is modified by homology methyltransferase RsmH^27–29^. In both mitoribosomes and *E. coli* ribosomes, m^4^C839 (of mitoribosomes) and m^4^C1402 (of *E. coli* ribosomes) modifications are located near the P-site codon of mRNA, which was speculated to play a role in fine-tuning P-site and increasing decoding fidelity^30^. Inactivation of human *METTL15* (or mouse *Mettl15*) perturbs the translation of mitochondrial protein-coding mRNAs and decreases mitochondrial respiration capacity^24–26^. Studies have reported that METTL15 may be highly correlated with childhood obesity^31^. Moreover, the assembly and stability of mitoribosomes also depend on mitochondrial ribosome-binding factors, such as hsRBFA. Acting as a scaffold protein, hsRBFA is recruited to mt-SSU 12 S rRNA helix 44 and helix 45, promoting proper decoration, assembly and maturation of mt-SSU^32^. Recent studies have shown that hsRBFA adapts a large conformational change, which ensures that TFB1M completes the dimethylation of helix 45 and then recruits METTL15 for further modification^33^. Our previous research has shown that hsRBFA recognizes 12 S rRNA via a novel binding mode. Binding of hsRBFA to 12 S rRNA was not only dependent on its KH-like domain but also dependent on the basic amino acid of its N-terminus, which effectively promotes the proper assembly of mitoribosomes^34^.

Understanding the molecular mechanisms that govern the interaction between METTL15, hsRBFA, and 12 S rRNA is critical for unraveling regulatory role of METTL15 in mitochondrial gene expression. In this study, we determined four crystal structures of the methyltransferase METTL15 in complex with different substrates. By analyzing these structures, we identified a series of key residues in METTL15 and hsRBFA that are responsible for specific recognition. Moreover, METTL15 recognized RNA substrates using the second scaffold-like domain (domain 2) through electrostatic interactions between the domain 2 and the sugar-phosphate backbone of RNA, indicating that hsRBFA might be the key factor in recruiting METTL15 to mt-SSU, instead of 12 S rRNA. Furthermore, knocking down METTL15 affected OXPHOS activity and cellular metabolic state, leading to increased ROS and membrane potential. Hence, through a combination of biochemical, structural, and functional approaches, we provided detailed understanding of the specific recognition mechanism underlying the interaction between METTL15, hsRBFA and 12 S rRNA, shedding a new light on the regulatory role of METTL15 in mitochondrial gene expression.

## Results

### Overall structure of human METTL15 in complex with SAM

**Fig. 1:**
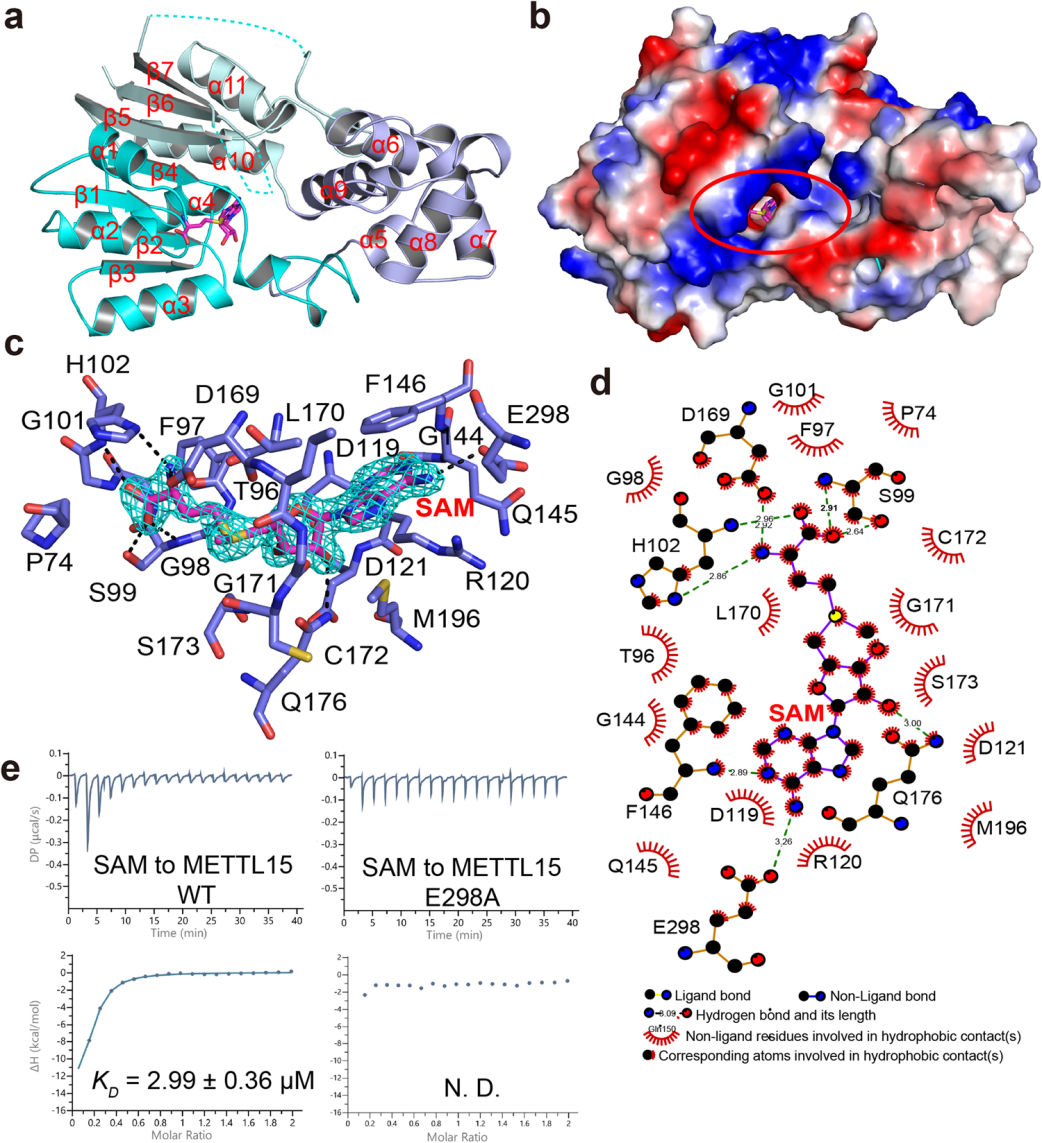
Structural overview of METTL15 in complex with SAM. **a** Cartoon representation of the METTL15–SAM binary complex. The two parts of MTases domain (domain 1) are colored in cyan and palecyan, the scaffold-like domain (domain 2) in slate and SAM in magenta, respectively. The missing loops of METTL15 are represented as cyan dotted lines. **b** The electrostatic potential of the METTL15–SAM complex is shown, in which positively charged, negatively charged and neutral areas are represented in blue, red and white, respectively. The SAM active pocket of METTL15 is highlighted as solid red circle. **c** The detailed interactions between METTL15 (slate) and the SAM (magenta). The *2Fo-Fc* electron-density map of SAM is contoured at 1.0 σ (gray). **d** Schematic representations of the recognition of SAM (colored purple and labeled in red) by METTL15 (colored bright orange and labeled in black). **e** The ITC fitting results of METTL15 WT and mutants by SAM.

Previous studies have revealed that METTL15 methyltransferase is responsible for the formation of the m^4^C839 residue of human mitochondrial 12 S rRNA^24–26^. To further investigate the mechanism of m^4^C839 modification, we determined the structures of apo METTL15 and the binary complex METTL15–SAM (Fig. 1a, b; Supplementary Fig. S1b). Comparison of these two structures revealed that the root-mean-square deviation (r.m.s.d.) of the Cα atom was only 0.145 Å (269 to 269 atoms). Therefore, we mainly used the METTL15–SAM complex for subsequent description. In the latter model, the METTL15–SAM complex included two molecules in an asymmetric unit both with observable electronic densities for SAM and most residues of METTL15, except for part of the loop (Asn^362^~Ala^371^ and Ser^384^~Gly^396^) (Fig. 1a). The detailed crystallographic statistics were summarized in Table 1.

**Table 1:** Data collection and refinement statistics.

Data collection	METTL15^70–407^ apo	METTL15^70–407^–SAM	METTL15^70–407^–RBFA^226–260^–SAM	METTL15^70–407^–slRNA1–RBFA^226–260^–SFG
Beamline	19U, SSRF	18U, SSRF	18U, SSRF	19U, SSRF
Space group	*P*3_1_21	*P*3_1_21	*P*2_1_2_1_2_1_	*C*222_1_
PDB code	8IPI	8IPK	8IPL	8IPM
Wavelength (Å)	0.9789	0.9789	0.9789	0.9789
Resolution (Å)	31.34-2.10 (2.14-2.10)^a^	25.25-1.90 (1.93-1.90)^a^	33.48-2.20 (2.28- 2.20)	33.53-3.10 (3.21-3.10)
Cell dimensions				
a, b, c (Å)	70.61, 70.61, 136.063	70.50, 70.50, 134.674	63.17, 70.98, 75.93	71.22 134.11 93.03
α, β, γ (°)	90, 90, 120	90, 90, 120	90, 90, 90	90, 90, 90
Unique reflections	23694 (1134)	31270 (1647)	16587 (789)	8204 (888)
Completeness (%)	100 (100)	99.9 (100)	92.61 (97.65)	97.42 (98.90)
Redundancy	17.9 (17.8)	15.0 (15.4)	8.9 (8.9)	9.0 (9.7)
I/σI	15.8 (2.8)	23.2 (2.2)	16.5 (6.2)	14.7 (3.7)
* R*_merge_ (%)	14.8 (80.0)	12.6 (100.3)	11.1 (50.7)	10.5 (61.6)
Refinement				
* R*_work_ (%)	19.32	18.23	17.99	25.25
* R*_free_ (%)	22.91	22.07	23.46	28.78
No. of atoms	2307			
Protein		2384	2373	2264
RNA				296
Water	110	113	32	
Average B factors (Å^2^)				
Protein	29.05	32.91	32.26	84.49
RNA				178.65
Water	31.19	32.43	27.32	
Root mean square deviations				
Bond lengths (Å)	0.007	0.006	0.008	0.002
Bond angles (°)	0.793	0.784	0.979	0.537
Ramachandran plot				
Favored (%)	97.67	98.36	97.66	97.29
Allowed (%)	2.33	1.64	2.34	2.71
Disallowed	0	0	0	0

^a^Values for the highest-resolution shell are shown in parentheses.

Similar to other members of rRNA methyltransferases, METTL15 has a two-domain structure: an MTase domain (domain 1) and a scaffold-like domain (domain 2). The domain 1 of METTL15 was slightly different from the conserved Rossman-like fold. The structure still folded with a central seven-stranded β-sheet flanked by five α-helices on each side, but the sequence was composed of two parts: the N-terminal sequence (Pro^74^ to Thr^179^) and the C-terminal sequence (Glu^295^ to Leu^407^) (Fig. 1a). The domain 2 of METTL15 contained five α-helices and one η-helix, which formed a scaffold for the binding of RNA substrates or other functional proteins (Fig. 1a). The SAM ligand fit snugly in the binding pocket with well-covered density (Fig. 1c). The conserved active pocket of METTL15 to accommodate SAM was surrounded by Thr^96^, Ser^99^, His^102^, Asp^119^, Arg^120^, Gln^145^, Phe^146^, Asp^169^, Ser^173^, Gln^176^, Glu^298^, and etc., which established hydrogen bonds, salt bridges and hydrophobic interactions with SAM (Fig. 1c, d). The base of SAM was sandwiched between Arg^120^ and Phe^146^, with its adenine ring packed with the aromatic ring of Phe^146^ via favorable π–π interactions. Additionally, the adenine ring formed multiple hydrogen bonds with the backbone nitrogen of Arg^120^, Phe^146^, and the carbonyl group of Glu^298^. Two hydroxyl groups in the ribose moiety of SAM formed hydrogen bonds with the side chains of Asp^119^ and Gln^176^, respectively. Finally, the carboxyl group of SAM formed several hydrogen bonds with Thr^96^, Ser^99^, His^102^ and Asp^169^. The above residues involved in SAM binding (either their sequences or their structures) were conserved from prokaryotes to eukaryotes (Supplementary Fig. S1a). Comparison of the electrostatic potential surface of these two structures showed that the positive charge at the SAM-binding pocket of METTL15 was significantly enriched after SAM binding (Fig. 1b; Supplementary Fig. S1b). Then, we mutated several residues of METTL15 SAM pocket (D119A/R120A, D169A and E298A) and employed isothermal titration calorimetry (ITC) assays to evaluate their contributions to SAM binding. The affinity of wild-type (WT) METTL15 for SAM was in the micromolar range (2.99 ± 0.36 μM) (Fig. 1e; Table 2). All these mutants weakened the interactions of METTL15 with SAM, particularly D119A/R120A and E298A, which almost eliminated the association capacity (Fig. 1e; Table 2; Supplementary Fig. S1c).

**Table 2:** The thermodynamic parameters of the ITC experiments.

METTL15^70–407^	RBFA	SAM	ΔH kcal/mol	-TΔS kcal/mol	*K*_*D*_ μM
METTL15_WT_	/	SAM	–17.20 ± 1.82	9.78	2.99 ± 0.36
METTL15_D119A/R120A_	/	SAM			N. D.
METTL15_D119A_	/	SAM	–31.70 ± 6.14	25.3	17.30 ± 0.31
METTL15_E298A_	/	SAM			N. D.
METTL15_WT_	RBFA^226-260^_WT_	/	–12.80 ± 0.11	4.42	0.62 ± 0.03
METTL15_WT_	RBFA^226-276^_WT_	/	–14.90 ± 0.27	6.44	0.46 ± 0.08
METTL15_WT_	RBFA^226-260^_C237A_	/			Weak binding
METTL15_WT_	RBFA^226-260^_H241A_	/	–14.90 ± 0.55	8.22	11.30 ± 0.85
METTL15_WT_	RBFA^226-260^_N245A_	/	–10.60 ± 0.20	2.26	0.61 ± 0.09
METTL15_WT_	RBFA^226-260^_Q247A_	/	–11.50 ± 0.20	2.92	0.41 ± 0.07
METTL15_WT_	RBFA^226-260^_I248A_	/			Weak binding
METTL15_WT_	RBFA^226-260^_Y251A_	/			Weak binding
METTL15_WT_	RBFA^226-260^_K252A_	/	–17.20 ± 3.10	10.90	19.60 ± 6.48
METTL15_WT_	RBFA^226–260^_Y251A/K252A_	/			N. D.
METTL15_WT_	RBFA^226–260^_H241A/Y251A/ K252A_	/			N. D.
METTL15_D209A_	RBFA^226-260^_WT_	/	–7.43 ± 0.32	0.583	7.94 ± 0.07
METTL15_N212A_	RBFA^226-260^_WT_	/	–9.50 ± 0.34	1.51	1.12 ± 0.03
METTL15_D215A_	RBFA^226-260^_WT_	/			Weak binding
METTL15_F291A_	RBFA^226-260^_WT_	/			Weak binding
METTL15_D209A/F291A_	RBFA^226-260^_WT_	/			N. D.
METTL15_D209A/N212A/D215A_	RBFA^226-260^_WT_	/			N. D.

*K*_*D*_ dissociation constant, ΔH binding enthalpy, -TΔS binding entropy, N. D. Not Detected. Each *K*_*D*_ value is presented as fitted value ± error.

### The C-terminal helix of hsRBFA is essential for METTL15 recognition

**Fig. 2:**
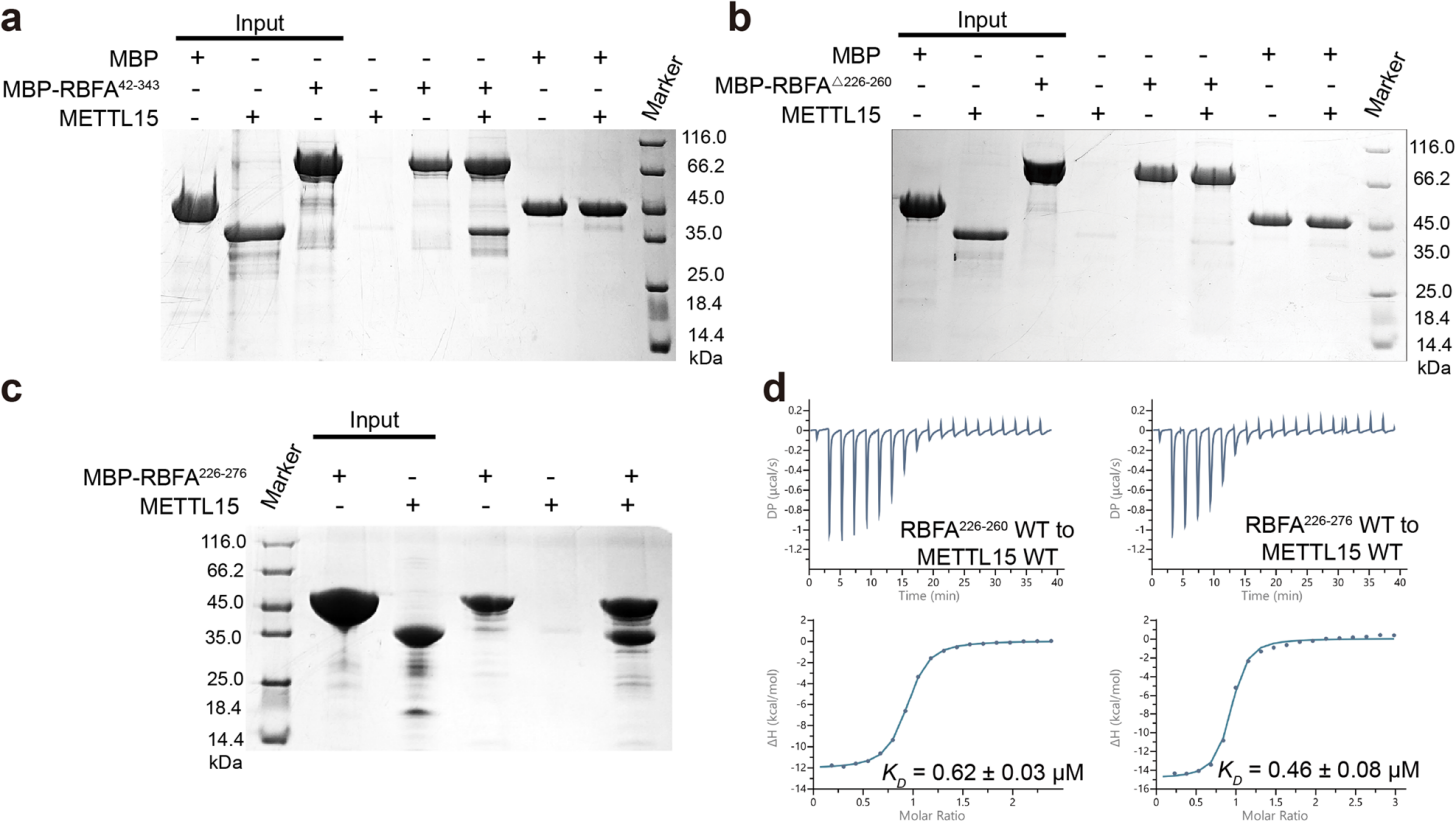
METTL15 recognized the C-terminal helix of hsRBFA. **a**–**c** Interactions of different MBP-tagged hsRBFA constructs with METTL15 visualized by Coomassie blue staining. The indicated MBP-hsRBFA fusion proteins or MBP alone were incubated with METTL15. The complexes were collected with glutathione-agarose resin and bound proteins were eluted and then subjected to SDS-PAGE. MBP or MBP-hsRBFA fusion proteins without METTL15 are shown as a negative control. **d** The ITC fitting results of WT METTL15 by the C-terminal helix of hsRBFA.

As a mitochondrial ribosomal assembly factor, hsRBFA binds to METTL15, which promotes further rRNA maturation and a large conformational change in hsRBFA^25,33^. We validated the recognition of hsRBFA by METTL15^70–407^ using pull-down experiments with various hsRBFA constructs in vitro. The results showed that the MBP-hsRBFA^42–343^ could interact with METTL15^70–407^, but the interaction was abolished in MBP-hsRBFA^△226–260^ (Fig. 2a, b; Supplementary Fig. S2a). Further pull-down assays revealed that the C-terminal helix of hsRBFA^226–276^ was responsible for METTL15 recognition (Fig. 2c; Supplementary Fig. S2a). Moreover, according to the ITC assays, the *K*_*D*_ values of METTL15 with hsRBFA^226–276^ and hsRBFA^226–260^ were 0.46 ± 0.08 μM and 0.62 ± 0.03 μM, respectively (Fig. 2d).

To further characterize the interactions between METTL15 and the C-terminal helix of hsRBFA, we crystallized and determined the complex structure of METTL15^70–407^ with SAM and hsRBFA^226–260^. The METTL15^70–407^–hsRBFA^226–260^–SAM ternary complex structure was subsequently refined to a resolution of 2.2 Å in space group *P 2*_*1*_. The crystal structure was solved by molecular replacement using the structure of apo METTL15^70–407^ solved previously as the search model. Finally, the *R*_*work*_ and *R*_*free*_ of the ternary complex structure were refined to 17.99% and 23.46%, respectively. The detailed crystallographic statistics were summarized in Table 1.

**Fig. 3:**
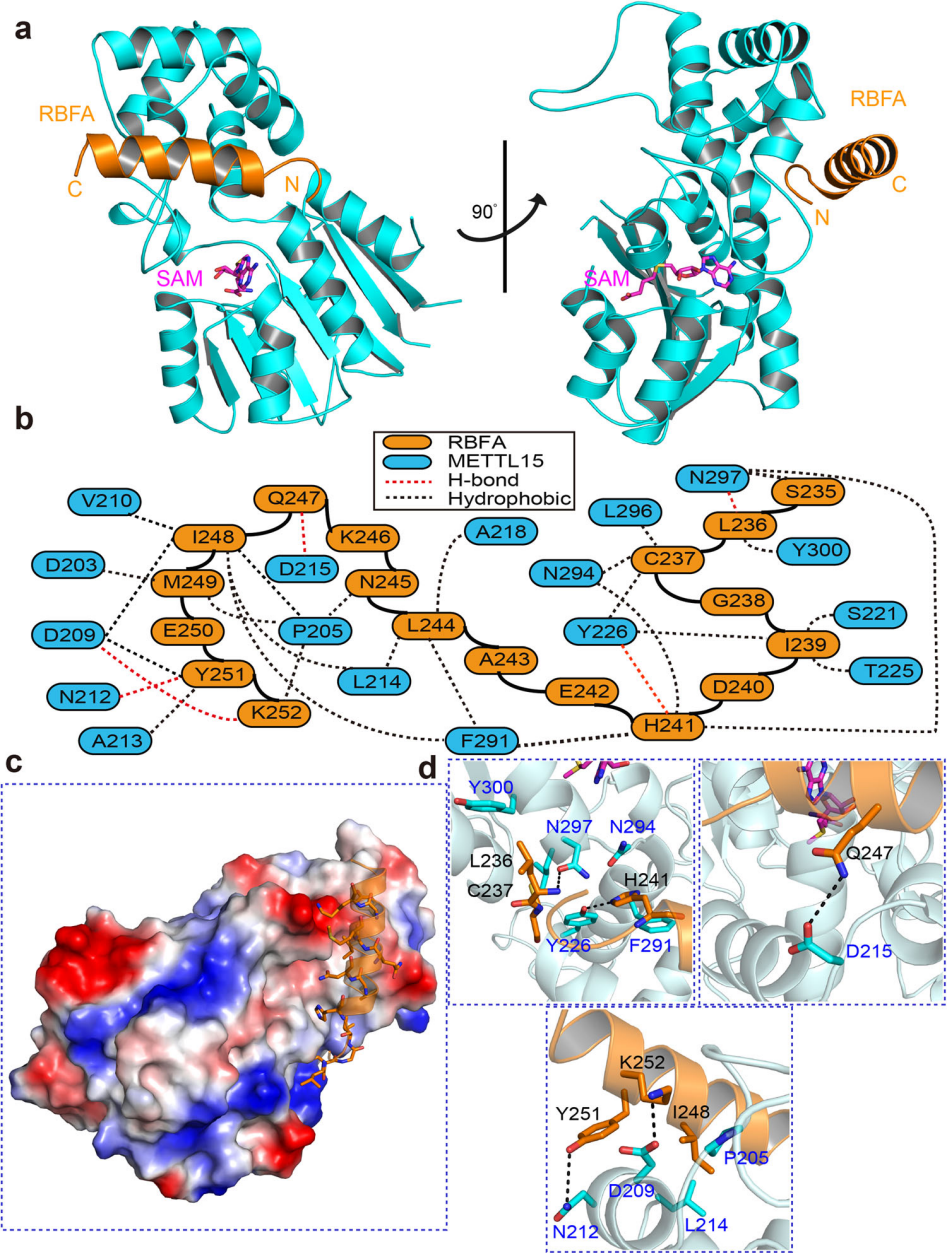
Structure of METTL15 in complex with hsRBFA^226–260^ and SAM. **a** Cartoon representation of the METTL15^70–407^–hsRBFA^226–260^–SAM ternary complex in two orientations related by a 180° rotation around a vertical axis. The asymmetric unit consists of the METTL15 (cyan), hsRBFA (bright orange) and SAM (magenta). **b** Schematic of hsRBFA interactions with METTL15, colored as described in (**a**). The red and black dotted lines indicate contacts mediated by hydrogen bonds and water-bridged hydrogen bonds, respectively. **c** The C-terminal helix of hsRBFA are represented as sticks on the molecular face of METTL15. The positively charged, negatively charged and neutral areas are represented in blue, red and white, respectively. **d** Higher magnification views of individual interactions between METTL15 (cyan, labeled blue) and hsRBFA (bright orange, labeled black). Hydrogen bonds are indicated with black dotted lines.

In the ternary structure, METTL15 formed a 1:1:1 complex with SAM and hsRBFA^226–260^ in an asymmetric unit (Fig. 3a). METTL15 and SAM in this ternary complex adopted a similar conformation with that in the METTL15–SAM complex, displaying an overall r.m.s.d. for Cα atoms of 0.361 Å. Most of the hsRBFA^226–260^ (Leu^236^~Asp^255^) were visible in the electron density map (Supplementary Fig. S2c), forming as a helix and lying on the side of METTL15 domain 2 surface (Fig. 3a, c). Superposition of the METTL15^70–407^–hsRBFA^226–260^–SAM ternary complex and the METTL15–SAM complex showed that the η-helix (Thr^339^~Glu^361^) of METTL15 domain 1 posed a physical barrier with hsRBFA helix and was not observed in the ternary complex (Supplementary Fig. S2e), indicating that the η-helix of METTL15 domain 1 is flexible, which may undergo a conformational change as a switch when METTL15 binds to hsRBFA.

The interactions between METTL15 and hsRBFA^226–260^ were shown in Fig. 3b, including hydrogen-bonding interactions and hydrophobic interactions. The helix of hsRBFA spanned one hydrophobic surface of METTL15 (Fig. 3c). The side chains of hsRBFA Cys^237^, Ile^239^, His^241^, Asn^245^ and Ile^248^ inserted into several wide and shallow hydrophobic pockets of METTL15, resulting in specific recognition between METTL15 and hsRBFA (Fig. 3c; Supplementary Fig. S2d). Moreover, the main chain of hsRBFA Leu^236^ formed a hydrogen bond with the peptide carbonyl group of METTL15 Asn^297^ (Fig. 3d). The side chain of hsRBFA His^241^ protruded into the aromatic cage surrounded by METTL15 Tyr^226^, Phe^291^, Asn^294^, and Asn^297^, and formed a direct hydrogen bond with the carbonyl of Phe^291^ side chain (Fig. 3d). The hydroxyl group of hsRBFA Gln^247^ side chain and carbonyl group of hsRBFA Tyr^251^ side chain formed specific hydrogen bonds with the carbonyl group of METTL15 Asp^215^ side chain and hydroxyl group of the side chain of METTL15 Asn^212^, respectively (Fig. 3d). HsRBFA Lys^252^ contacted the side chain of METTL15 Asp^209^ via the positively charged side chain (Fig. 3d).

To confirm the specific recognition between METTL15 and hsRBFA^226–260^ in vitro, we introduced alanine mutations into METTL15 and hsRBFA^226–260^, respectively. Subsequently, we performed ITC assays to measure the binding affinities between WT METTL15 and hsRBFA^226–260^ mutants or METTL15 mutants and WT hsRBFA^226–260^ (Table 2; Supplementary Figs. S3, S4). As expected, the introduction of H241A, Y251A and K252A single mutations on hsRBFA^226–260^ resulted in more than 10-fold decrease in the binding affinities to hsRBFA^226–260^ with METTL15 (*K*_*D*_ = 11.30 ± 0.85 μM, 11.30 ± 0.85 μM and 11.30 ± 0.85 μM, respectively), compared with 0.62 ± 0.03 μM for WT hsRBFA^226–260^ (Supplementary Fig. S3). HsRBFA^226–260^ C237A and I248A mutants also exhibited substantial reduction in binding affinities to METTL15 (Supplementary Fig. S3). It is not surprising that hsRBFA^226–260^ Y251A/K252A and H241A/Y251A/K252A mutations abolished the binding to METTL15 completely (Supplementary Fig. S3). Similarly, METTL15 single mutations (D209A, D215A and F291A) notably decreased the binding affinities between METTL15 and hsRBFA^226–260^ (Supplementary Fig. S4). Moreover, METTL15 double mutation (D209A/F291A) or triple mutation (D209A/N212A/D215A) also abolished hsRBFA^226–260^ binding (Supplementary Fig. S4). Circular dichroism (CD) spectral analyses confirmed that all mutants of *METTL15* and hsRBFA^226–260^ maintained a secondary structure composition similar to that of the WT proteins.

### METTL15 recognizes RNA substrates by electrostatic interactions in vitro

**Fig. 4:**
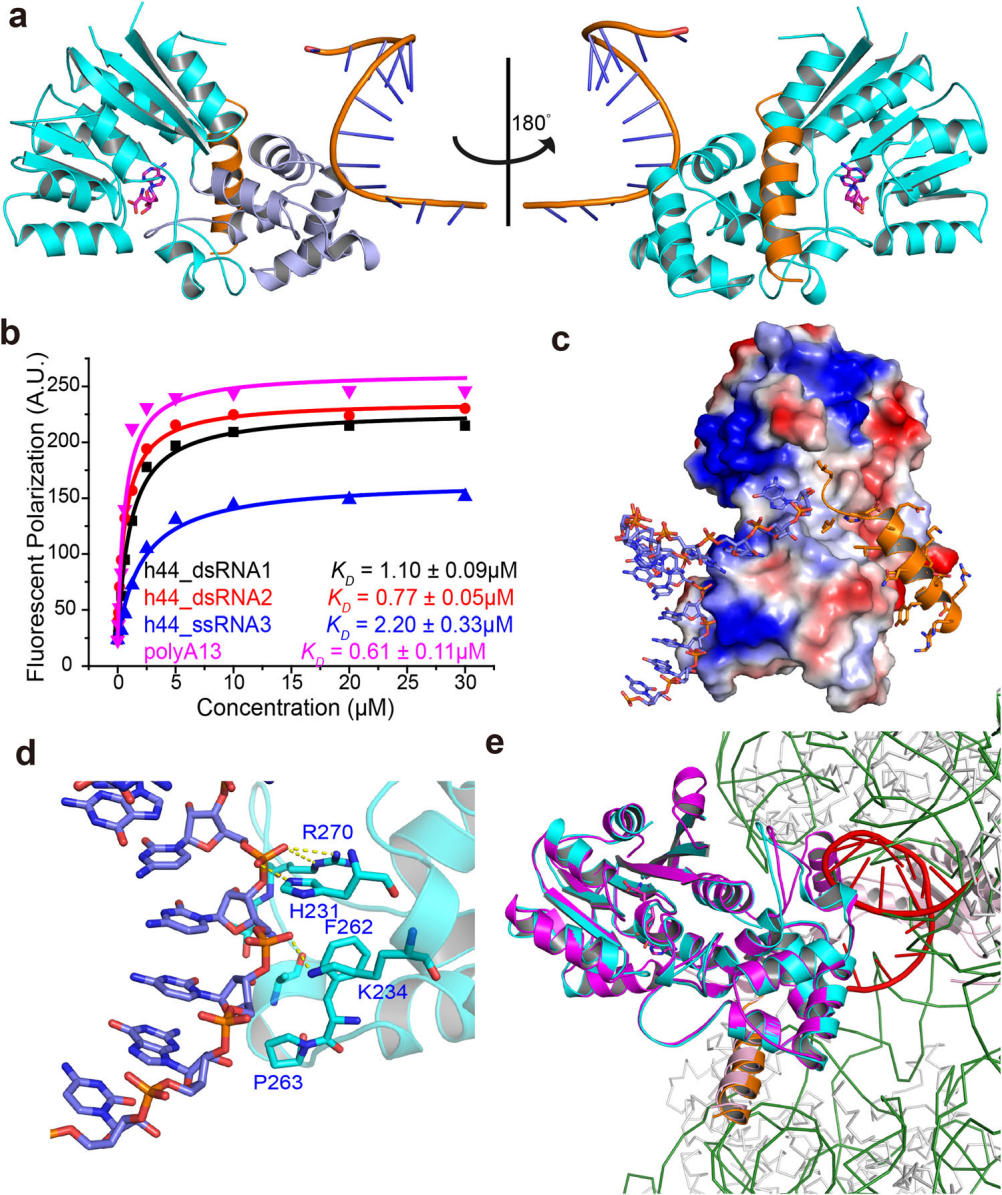
Structure of METTL15 in complex with RNA, hsRBFA^226–260^ and SFG. **a** Cartoon representation of the METTL15^70–407^–RNA–hsRBFA^226–260^–SFG quaternary complex in two orientations related by a 180° rotation around a vertical axis. The asymmetric unit consisted of the METTL15 (cyan), h44_dsRNA1 (slate), hsRBFA (bright orange) and SFG (magenta). In the left pannel, the MTases domain (domain 1) is colored in cyan and the scaffold-like domain (domain 2) in slate, respectively. **b** The binding affinities of 5’-FAM-labeled RNAs for METTL15 determined by FP experiments are shown. **c** The RNA and C-terminal helix of hsRBFA are represented as sticks binding to the either side of METTL15, respectively. The positively charged, negatively charged and neutral areas are represented in blue, red and white, respectively. **d** Higher magnification views of individual interactions between METTL15 (cyan, labeled blue) and h44_dsRNA1 (slate). Hydrogen bonds are indicated with yellow dotted lines. **e** Superposition of the METTL15^70–407^–RNA–hsRBFA^226–260^–SFG quaternary complex with the cryo-electron structure of pre-mt-SSU (PDB ID 7PNX). The METTL15^70–407^–RNA–hsRBFA^226–260^–SFG quaternary complex colored as described in (**a**), except the h44_dsRNA1 colored red. The METTL15 and hsRBFA in pre-mt-SSU are colored magenta and light pink, respectively.

To further explore the RNA substrates, we employed fluorescent polarization (FP) assays to test a series of RNAs for their binding affinities to METTL15. METTL15 bound to dsRNA (h44_dsRNA1) or ssRNA (h44_ssRNA3) derived from h44 around the 12 S rRNA m^4^C839 residue, with *K*_*D*_ values of 1.10 ± 0.09 μM and 2.20 ± 0.33 μM, respectively (Fig. 4b). Interestingly, the GAAG tetraloop substitution of the native loop in dsRNA (h44_dsRNA2) didn’t affect the binding ability between METTL15 and RNA (Fig. 4b; Supplementary Fig. S5a). Additionally, METTL15 could effectively bind to the ssRNA (polyA13) with non-sequence-specificity (Fig. 4b). Furthermore, adding hsRBFA^226–260^ didn’t affect the binding affinities between METTL15 and RNA substrates (Table 3).

**Table 3:** Binding affinities of WT and mutant METTL15 proteins to different RNA substrates by FP assays.

Protein	RBFA	RNA	K_d_ (μM)
METTL15 (wildtype)	/	h44_dsRNA1	1.10 ± 0.09
wildtype	/	h44_dsRNA2	0.77 ± 0.05
wildtype	/	h44_ssRNA3	2.20 ± 0.33
wildtype	/	h44_dsRNA4	2.33 ± 0.13
wildtype	/	polyA13	0.61 ± 0.11
wildtype	+RBFA	h44_dsRNA1	1.15 ± 0.08
wildtype	+RBFA	h44_ssRNA3	1.76 ± 0.16
H231A/K234A/F262A	/	h44_dsRNA1	N. D.
F262A/P263A/K270A	/	h44_dsRNA1	N. D.
R327A/K330A/R331A	/	h44_dsRNA1	1.03 ± 0.15
K380A/K381A	/	h44_dsRNA1	2.68 ± 0.29

We then determined the binding mode between METTL15 and the RNA substrate (h44_dsRNA1 in Supplementary Fig. S5a), together with hsRBFA^226–260^ and S-adenosyl-methionine analog sinefungin (SFG). The METTL15^70–407^–RNA–hsRBFA^226–260^–SFG quaternary complex structure was subsequently refined to a resolution of 3.1 Å in space group *C222*_*1*_. The crystal structure was solved by molecular replacement using the structure of ternary complex solved before as the search model. Finally, the *R*_*work*_ and *R*_*free*_ of the quaternary complex structure were refined to 25.25% and 28.79%, respectively. The detailed crystallographic statistics were summarized in Table 1.

In the structure of the quaternary complex (Fig. 4a), METTL15–RNA–hsRBFA^226–260^–SFG adopted a similar conformation to that in the METTL15^70–407^–hsRBFA^226–260^–SAM ternary complex, displaying an overall r.m.s.d. for Cα atoms of 0.475 Å. The helix of hsRBFA^226–260^ and the RNA substrate bound on either side of METTL15 domain2 (Fig. 4c). Unexpectedly, both the 5’ end and the 3’ end of h44_dsRNA1 bases in the quaternary complex partially paired with the neighboring RNA in the next symmetry equivalent for better crystallization, respectively (Supplementary Fig. S5b, c).

Consistent with the previous FP assays, METTL15 interacted with the phosphodiester backbone of the RNA instead of the bases (Fig. 4d). The residues in the N-terminus of helix α7 and in the loop between helices α8 and α9 of METTL15 formed a positively charged channel to accommodate and recognize the RNA substrate. METTL15 His^231^, Lys^234^ and Arg^270^ formed several direct hydrogen bonds with the phosphodiester backbone of the RNA mainly via the positively charged side chain, while Phe^262^ and Pro^263^ recognized the phosphodiester backbone of the RNA via the hydrophobic interactions (Fig. 4d). The side chains of Pro^263^, Ala^266^ and Thr^269^ recognized the ribose of the RNA via the hydrophobic interactions (Fig. 4d). Furthermore, the introduction of H231A/K234A/F262A or F262A/P263A/K270A mutations to *METTL15* abolished the binding affinities to RNA substrate (Table 3). The electrostatic potential of the surface of METTL15 showed that domain1 of METTL15 also contained a positively charged region, which was reported to recognize the h24 of 12 S rRNA in the cryo-electron structures of pre-mt-SSU (PDB ID 7PNX, 7PNY and 7PNZ)^33^ (Fig. 4c, e). However, mutants R327A/K330A/R331A or K380A/K381A with key residues of this region being replaced showed comparable RNA binding affinities to WT *METTL15* (Table 3). Therefore, METTL15 domain 2 plays a key role in RNA substrate recognition.

Taken together, our biochemical and structural results showed that METTL15 recognizes RNA substrates without sequence specificity in vitro, which indicated the binding between METTL15 and its RNA substrate was contributed by the electrostatic interactions. HsRBFA, instead of RNA, might be the key factor in recruiting METTL15 to mt-SSU for m^4^C839 modification.

### METTL15 knockdown caused significantly altered cellular metabolic state

**Fig. 5:**
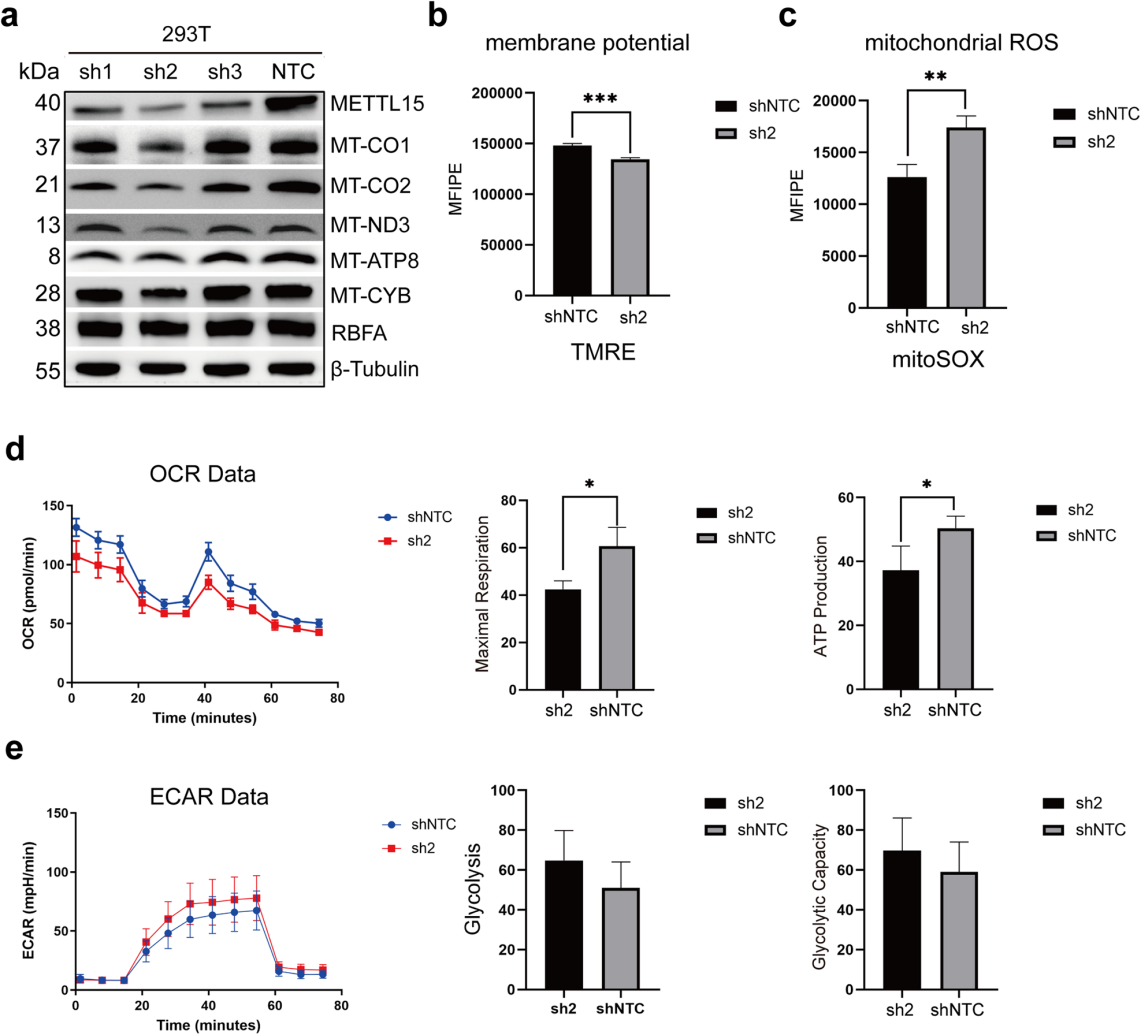
Effect of METTL15 knockdown on mitochondrial functions. **a** Western blot analyzing protein levels of METTL15 and mitochondrial gene-encoded proteins in OXPHOS complexes in HEK293T cells transfected with different shRNAs. β-tubulin served as a loading control. NTC, non-targeting control. **b** Flow cytometry analyzing mitochondrial membrane potential in HEK293T cells. The mean and standard deviation of mean fluorescence intensity (MFI) for three repetitions are shown as bars. shNTC, non-targeting control. **c** Flow cytometry analyzing mitochondrial reactive oxygen species. The mean and standard deviation of MFI for three repetitions are shown as bars. **d** Seahorse analyzing oxidative phosphorylation levels upon METTL15 knockdown. **e** Seahorse analyzing glycolysis upon METTL15 knockdown.

As our in vitro assays revealed the recognition mode between METTL15, hsRBFA and RNA, we tried to further study the biological function of METTL15 and hsRBFA on mitochondria in HEK293T cells. We knocked down METTL15 in HEK293T cells using three different shRNAs (sh1, sh2 and sh3, Supplementary Table S1), among which sh2 downregulates the levels of METTL15 mRNA and protein by more than 70%, according to the qRT-PCR and western blotting results (Fig. 5a; Supplementary Fig. S6a). Moreover, m^4^C in bacteria, mediated by the *E. coli* homologous methyltransferase RsmH may play a role in fine-tuning ribosomal decoding center and increasing decoding fidelity^30^. Therefore, we explored the impact of METTL15 knockdown on the translation of protein components of OXPHOS system encoded by the mitochondrial genome. The results showed that mitochondrial gene-encoded protein (mt-protein) expressions were decreased upon METTL15 knockdown, especially in the sh2 group. Furthermore, to investigate whether the key residues we identified previously influenced the mt-protein translation in vivo, we expressed different METTL15 mutants in cells to rescue deficiency of METTL15 induced by knockdown (Supplementary Fig. S6b). The results were consistent with our in vitro studies showing that elimination of the interactions between METTL15 with either hsRBFA or RNA affected mt-protein translation.

Besides, knockdown of METTL15 might lead to dysfunction of OXPHOS complex by impacting functions of the proteins translated in mitochondria, which results in malfunction of proton pump gradients across inner mitochondrial membrane and imbalance of the ROS. Therefore, we monitored the mitochondrial membrane potential and ROS levels by flow cytometry (Supplementary Figs. S6c, d). The membrane potential strikingly decreased, while ROS were increased in METTL15 knockdown cells (Fig. 5b, c). Furthermore, the levels of mitochondrial OXPHOS and glycolysis were detected using the Seahorse real-time cell metabolic analysis. The level of OXPHOS decreased, while the level of glycolysis increased (Fig. 5d, e), which indicated that cellular metabolic state in METTL15 knockdown cells was changed.

**Fig. 6:**
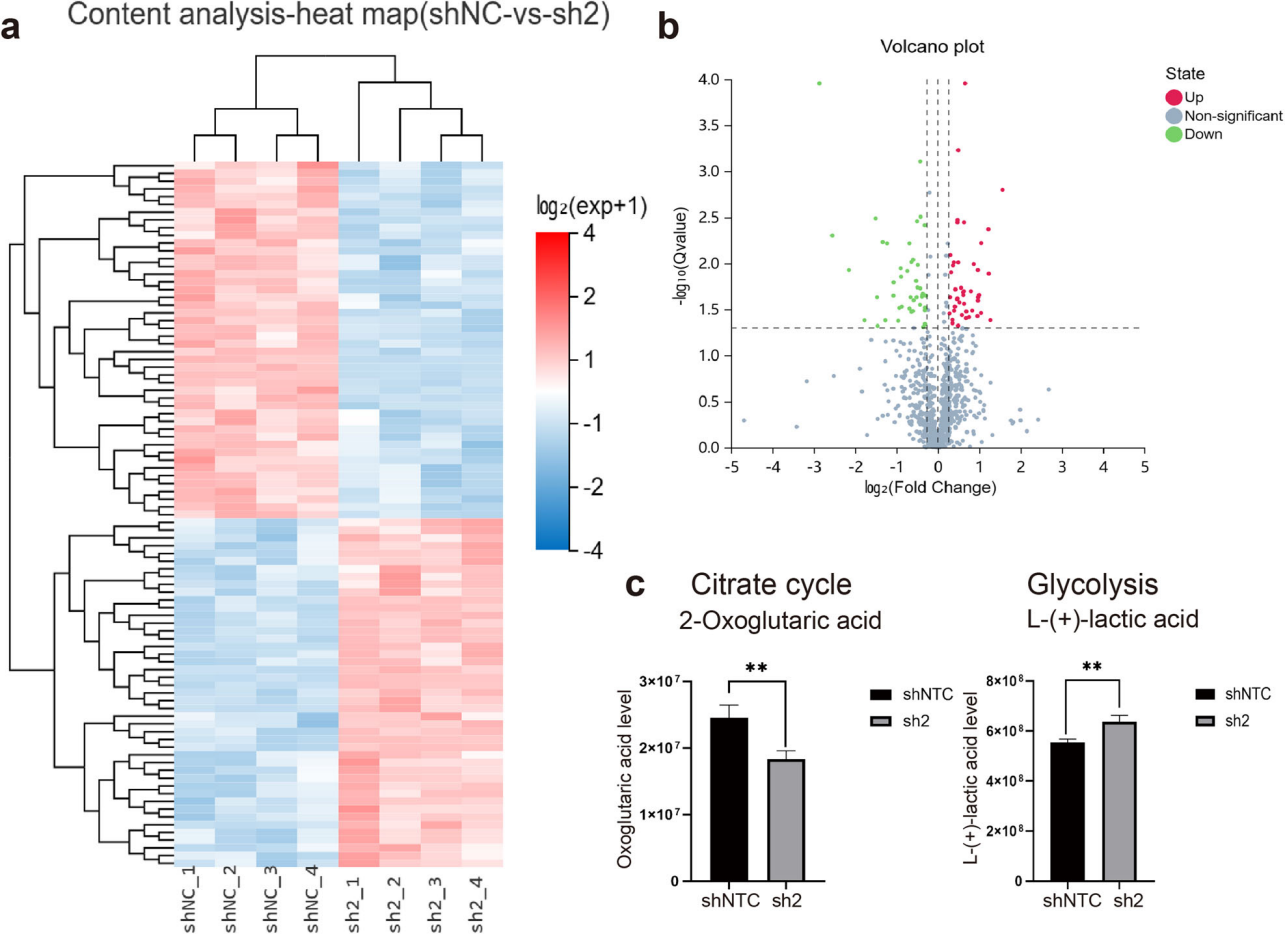
METTL15 knockdown alters cellular metabolic state. **a** 91 significantly differential metabolites (*q* value < 0.05) between shNC cells and METTL15 sh2 knockdown cells are shown in the heatmap. **b** The volcano diagram shows different metabolites of the two groups. (Fold change ≥ 1.2 or Fold change ≤ 0.83). **c** Two metabolites implicated in TCA cycle and glycolysis are compared and shown as histograms.

To determine the cellular metabolic alterations in METTL15 knockdown cells, we applied untargeted metabolomic analysis to METTL15 knockdown cells and used the shNC cells as a control. All samples were processed and analyzed using UPLC-MS/MS following the standardized protocol. In total, 3,525 metabolite peaks were identified, among which 1075 metabolites were identified based on MS/MS spectra through the BGI reference library, MzCloud, KEGG and HMDB (Supplementary Table S3), with coefficients of relative standard deviation < 0.30 across quality control (QC) samples. 91 out of 1075 metabolites were significantly associated with METTL15 knockdown (*q* value < 0.05) (Fig. 6a). Compared to control cells, the volcano plots showed that 45 metabolites were significantly increased (fold change ≥ 1.2) and 46 were decreased (fold change ≤ 0.83) in METTL15 knockdown cells (Fig. 6b). Consistent with our Seahorse real-time cell metabolic analysis, the metabolites were implicated in the decreased citrate cycle and increased glycolysis (2-oxoglutaric acid and L-(+)-lactic acid), respectively (Fig. 6c), which indicated a metabolic switch from mitochondrial OXPHOS to aerobic glycolysis. The level of lactate secretion increased ~1.3-fold in METTL15 knockdown cells compare with WT cells, which was more obvious in *METTL15* KO cells as studied by previous study^26^. As reported, the increase in intracellular metabolite lactate might raise the level of lactylation modification of certain proteins in the nucleus or cytoplasm, which in turn regulates the transcription and expression of related genes^35–37^. To explore the effect of elevated lactate secretion in METTL15 knockdown cells, we tested the level of several histone lactylation in METTL15 knockdown cells and WT cells, respectively. The western blotting results showed that levels of H4K12-lactylation (H4K12-la) and H3K9-lactylation (H3K9-la) were increased in METTL15 knockdown cells (Supplementary Fig. S7a). Moreover, metabolic pathway enrichment analysis of differentially abundant metabolites based on KEGG database showed that in METTL15 knockdown cells, many metabolic pathways were changed (Supplementary Fig. S8a). Some metabolites involved in pathways such as tricarboxylic acid (TCA) cycle, pyruvate metabolism, D-Glutamine and D-glutamate metabolism, D-arginine and D-ornithine metabolism, were dysregulated upon METTL15 knockdown (Fig. 6c; Supplementary Fig. S8b). Taken together, a proper function of METTL15 is critical to mitoribosome biogenesis and cellular metabolic state maintenance.

## Discussion

METTL15 was reported as a methyltransferase responsible for the m^4^C839 modification in the mt-SSU 12 S rRNA h44. A recent cryo-electron microscopy study on the structure of the mt-SSU suggested that hsRBFA promotes TFB1M binding and dimethylation of mt-SSU rRNA h45, which enables METTL15 binding and promotes further rRNA maturation^33^. In our study, we determined a series of crystal structures of METTL15 in complex with different substrates and analyzed specific interactions of METTL15 with SAM, hsRBFA and 12 S rRNA in detail. Since METTL15 recognizes RNA without sequence specificity, hsRBFA might be the key factor for METTL15 recruitment to mt-SSU. We also observed reduced amounts of mitochondrial genome-coding proteins, detected decreased membrane potential and increased ROS in METTL15 knockdown cells, accompanied by an abnormal cellular metabolic state. An increased level of glycolysis led to elevated lactate secretion (Fig. 6c), which induced the altered histone lactylation in the nucleus. Among the lactating sites, levels of H4K12-la and H3K9-la increased most significantly in METTL15 knockdown cells (Supplementary Fig. S7a). A recent study revealed that elevated histone H4K12-la is the most common form of histone lactylation in microglia of Alzheimer’s disease mice, which activates transcription of glycolytic genes and exacerbates microglial dysfunction in AD^38^. It was also found that lactate not only functions as a metabolic substrate to provide energy but can also functions as a signaling molecule to modulate cellular functions under pathophysiological conditions. Lactylation highlights the novel role of lactate in regulating transcription, cellular functions and disease pathogenesis^39^. Intriguingly, homozygous *Mettl15* KO mice survived and exhibited a much milder phenotype than *Tfb1m*^*−/−*^ mice, yet decreased exercise and learning capability were detected^40^. Furthermore, H3K9-la was recently identified to promote tumorigenicity of liver cancer stem^41^. More detailed studies exploring the regulation between METTL15 and histone lactylation are needed and *Mettl15* KO mice might be a suitable model.

The METTL15 ortholog RsmH is responsible for catalyzing m^4^C1402 of the SSU 16 S rRNA in *E. coli*. The alignment of our binary complex METTL15–SAM structure with the RsmH structure (PDB ID 3TKA) showed that the r.m.s.d. for Cα atoms of the two methyltransferases was 1.216 Å (210 to 210 atoms) (Supplementary Fig. S9a). The two structures shared highly similar conformations, except for some flexible loops. The key residues of active pocket to accommodate SAM were conserved between METTL15 and RsmH (Supplementary Fig. S1a). Strikingly, the alignment of our quaternary complex METTL15^70–407^–RNA–hsRBFA^226–260^–SFG structure with the RsmH structure showed that the helix of hsRBFA posed a physical barrier with the inactive cytidine in RsmH structure (Supplementary Fig. S9b, indicated with a red circle). As we described before, the C-terminal helix of hsRBFA is essential for METTL15 incorporation into mt-SSU. However, the C-terminal helix was apparently missing in RBFA of *E. coli* (Supplementary Fig. S9c), indicating that the incorporation mechanisms of METTL15 and RsmH into small ribosomal subunits are quite different.

Notably, METTL17, another METTL family member located in mitochondria, was also reported to affect levels of m^4^C839 and m^5^C841 modifications in 12 S mt-rRNA^42^. Likewise, inactivation of METTL17 also resulted in loss of OXPHOS activity and affected cellular metabolome in mouse embryonic stem cells. Nathan J. Harper, *et al*. recently reported a linear assembly pathway of mt-SSU intermediates bound by METTL17, in which METTL17 acts as an assembly factor and mediates the formation and compaction of the head domain of mt-SSU intermediates^43^. Moreover, METTL17 binds to mt-SSU rRNA h31 and prevents premature association of METTL15. Then, METTL15 outcompetes METTL17 to complete the final modification of the decoding center. *METTL17* KO leads to an immature head domain of intermediates, failing to recruit METTL15, further showing reductions in m^4^C839 and m^5^C841 of 12 S mt-rRNA^43^. However, METTL17 is not just as an assembly factor, its function is dependent on its SAM-binding ability, as the SAM-binding-deficient mutant of METTL17 fails to rescue the decreased level of m^4^C839 12 S mt-rRNA caused by *METTL17* KO^42^. Therefore, how does METTL17 function as an enzyme during mt-SSU assembly? Is there a functional correlation between METTL15 and METTL17 during mt-SSU assembly? Or are alternative pathways present in the cell? The assembly of mt-SSU does not occur in a strictly linear manner^44^. In addition, in WT HAP1 cells, the m^4^C839 methylation level is only ∼14%, which is strikingly lower than that of other rRNA methylated sites in mitoribosomes^24,45–47^. We also found that protein expression level of METTL17 was slightly elevated in METTL15 knockdown cells (Supplementary Fig. S7b), implying a correlation between METTL15 and METTL17. We are currently working on the possible relevance between these two m^4^C methyltransferases.

The proper assembly and maturation of mitoribosomes are critical for a proper function of the mitochondria. rRNA and tRNA modifications play key roles in mitoribosome biogenesis and efficient and accurate protein translation, which are tightly regulated. The studies of mitoribosome assembly and RNA modification mechanisms have broad implications for understanding mitochondrion biology and the pathogenesis of mitochondrial diseases.

## Materials and methods

### Protein expression and purification

DNA encoding human METTL15 (residues 70–407) was amplified and incorporated into a modified pET28a (Novagen) plasmid. The modified pET-28a plasmid contained an N-terminal SUMO-tag and a ULP1 protease cleavage site. The protein was expressed in *E. coli* Rosetta (DE3) cells (Novagen) cultured in LB medium at 37 °C to OD_600_ = 1.0, and then shifted to 16 °C for 24 h after induction with 0.2 mM isopropyl-β-D-thiogalactopyranoside (IPTG). Bacterial pellets were resuspended in buffer A (50 mM Na_2_HPO_4_, 1 M NaCl, pH 7.5) and lysed by sonication on ice. The crude lysate was then centrifuged at 14,000 rpm for 30 min at 4 °C. The supernatant was applied to a Ni-NTA column (QIAGEN), followed by size exclusion chromatography using a Superdex 200 (GE Healthcare) column. After cleavage with ULP1 protease overnight at 16 °C to remove the SUMO-tag, an additional HiTrap SP FF column purification step was employed. The purified protein was concentrated to ~10 mg/mL in buffer B (50 mM Na_2_HPO_4_, 150 mM NaCl, pH 7.0) and stored at –80 °C. All mutants were generated using a MutanBEST kit (TaKaRa) and confirmed by DNA sequencing. The mutant proteins were purified using the protocol described above.

For maltose binding protein (MBP) pulldown assays, DNAs encoding human hsRBFA (residues 42–343, 42–201, 226–276 and 226–260) were amplified and incorporated into a modified pET22b (Novagen) plasmid, respectively. The modified pET-22b plasmid contained N-terminal 6× His and MBP tags. The 6× His-tagged MBP-hsRBFA fused protein was expressed in *E. coli* BL21-Gold (DE3) cells (Novagen) cultured in LB medium at 37 °C to OD_600_ = 0.8 ~ 1.0, and then shifted to 16 °C for 24 h after induction with 0.5 mM IPTG. Bacterial pellets were resuspended in buffer C (20 mM Tris, 1 M NaCl, pH 7.5) and lysed by sonication on ice. The crude lysate was then centrifuged at 14,000 rpm for 30 min at 4 °C. The supernatant was applied to a Ni-NTA column (QIAGEN), followed by size exclusion chromatography using a Superdex 200 (GE Healthcare) column. The purified hsRBFA protein was diluted in buffer D (20 mM Tris, 200 mM NaCl, pH 7.5). The mutant hsRBFA^△226–260^ was generated using a MutanBEST kit (TaKaRa) and confirmed by DNA sequencing. The mutant protein was purified using the protocol described above.

For crystallization and ITC assays, DNA encoding human hsRBFA (residues 226–276 or 226–260) was amplified and incorporated into a modified pGEX-4T-1 (Novagen) plasmid, in which the thrombin protease site was substituted for a tobacco etch virus (TEV) cleavage site. The proteins were expressed in *E. coli* BL21-Gold (DE3) cells (Novagen) cultured in LB medium at 37 °C till OD600 reaches ~1.0, then shifted to 16 °C and induced with 0.2 mM IPTG overnight. Bacterial pellets were resuspended in buffer C and lysed by sonication on ice. The fusion proteins were purified on glutathione-Sepharose beads (GE Healthcare) and eluted with buffer C containing 30 mM reduced L-glutathione. The GST tag was cleaved by TEV protease overnight at 16 °C, the protein was then purified by a Superdex 75 column (GE Healthcare). Finally, proteins were concentrated to ~15 mg/mL in buffer D and stored at –80 °C. All mutants were generated using a MutanBEST kit (TaKaRa) and confirmed by DNA sequencing. The mutant proteins were purified using the protocol described above.

### RNA preparation

For crystallization, RNA oligomers were purchased from Accurate Biotechnology (Hunan) Co.,Ltd, ChangSha, China and dissolved in diethyl pyrocarbonate (DEPC)-treated water to a final concentration of 1 mM. Prior to use, the RNA substrates were heat-denatured at 95 °C for 5 min and annealed on ice for 5 min. All RNA oligomers used in this study were listed in Supplementary Table S1.

### Pull-down assay

For the pull-down assay of the MBP-tagged or GST-tagged hsRBFA against the METTL15 protein, MBP-tagged hsRBFA (residues 42–343, 42–201, 226–276) or GST-tagged hsRBFA (residues 226–260) were bound to MBP beads or glutathione-Sepharose beads (GE Healthcare), respectively, and then the beads were incubated with METTL15 protein in buffer B for 2 h at 4 °C. After washing six times with buffer B containing 0.1% TritonX-100, the results were analyzed on Coomassie stained SDS-polyacrylamide gels.

### Protein crystallization, data collection and structure determination

Apo METTL15^70–407^ was concentrated to ~8 mg/mL in buffer B and crystallized in 10% PEG monomethyl ether 2000, 0.2 M ammonium sulfate, 0.1 M sodium acetate (pH 5.5) at 16 °C by vapor diffusion in sitting drops. METTL15^70–07^ and S-adenosyl methionine (SAM), were mixed at a 1:5 molar ratio at a final concentration of ~8 mg/mL. Crystals of the METTL15^70–407^–SAM complex were grown at 16 °C using the hanging drop vapor diffusion method by mixing 1 μL of mix with 1 μL reservoir buffer (42% PEG 600, 0.2 M imidazole malate, pH 5.5). METTL15^70–407^, hsRBFA^226–260^ and SAM were mixed at a 1:2:5 molar ratio, and incubated overnight at 4 °C. The ternary complex was crystallized in 17% w/v PEG 4000, 0.05 M Potassium chloride, 0.1 M Lithium chloride, 0.012 M Spermine tetrahydrochloride, 0.05 M MES pH 6.5 at 16 °C by vapor diffusion in sitting drops. METTL15^70–407^, hsRBFA^226–260^, RNA substrate and S-adenosyl methionine analog (sinefungin, SFG) were mixed at a 1:2:1.2:5 molar ratio, and incubated overnight at 4 °C. The quaternary complex was crystallized in 20% PEG 4000, 0.2 M ADA (pH 6.4) at 16 °C by vapor diffusion in sitting drops.

Crystals were soaked in mother liquor supplemented with 20% glycerol before being flash-frozen in liquid nitrogen. X-ray diffraction data for the crystals were collected on beamline 19U of the Shanghai Synchrotron Radiation Facility (SSRF). The data were processed with HKL2000 and programs in the CCP4 suite. The apo structure of the METTL15^70–407^ was solved by molecular replacement with the program MOLREP^48^, using AlphaFold model of METTL15 as the search model (AF-A6NJ78-F1). The apo METTL15^70–407^ structure was then recruited as the search model in the molecular replacement using program MOLREP for the structures of METTL15^70–407^–SAM complex, METTL15^70–407^–hsRBFA^226–260^–SAM ternary complex and METTL15^70–407^–hsRBFA^226–260^–RNA–SFG quaternary complex. The hsRBFA peptide was then modeled in COOT^49^. All initial models were refined using the maximum likelihood method implemented in REFMAC5^50^ as part of CCP4i program suite and rebuilt interactively using the program COOT. Final refinement strategies included XYZ coordinates, individual B-factors, occupancies, and automated correction of N/Q/H errors using PHENIX^51^. Crystallographic parameters were listed in Table 1. All images of the structures were prepared using PyMOL (http://www.pymol.org/).

### CD measurements

Far-UV CD spectra of METTL15 and its mutants were determined using an Applied Photophysics Chirascan spectrometer at 298 K. The spectra were recorded at wavelengths ranging from 195 to 260 nm using a 0.05 cm path length cell. The protein samples were diluted to 0.1 mg/mL with the buffer B. A buffer-only reference was subtracted from each curve. All samples were tested in triplicate.

### ITC assays

ITC assays were performed on a Microcal PEAQ-ITC instrument (Malvern) at 20 °C. The concentrations of proteins were determined spectrophotometrically. HsRBFA and METTL15 proteins were dialyzed against buffer B and adjusted to 600 μM and 40 μM, respectively. SAM and METTL15 proteins were dialyzed against buffer B and adjusted to 500 μM and 50 μM, respectively. Thermodynamic data were analyzed with a single binding site model using MicroCal PEAQ-ITC Analysis Software provided by the manufacturer. The thermodynamic parameters of the ITC experiments were listed in Table 2.

### FP assays

Different lyophilized 5’-FAM (carboxyfluorescein)-labeled RNA oligomers were purchased from Accurate Biotechnology (Hunan) Co.,Ltd, ChangSha, China, dissolved in DEPC-treated water to a final concentration of 100 μM and stored at –80 °C. The stock (100 μM) was diluted to 80 nM in dilution buffer B. All RNA oligomers used in this study were listed in Supplementary Table S1. Equilibrium dissociation constants of different RNAs with METTL15 and its mutants were determined by measuring FP, as previously described. METTL15 and its mutants were first diluted to 20 times the highest concentration used in the binding system, and then successively diluted 2-fold until the lowest desired concentration was reached. Before the assay, 100 μL of 80 nM fluorescence-labeled RNA was mixed with 100 μL of protein stocks from the diluted series and incubated for 15 min. Samples were then excited at 485 nm, and FP was detected at 525 nm using a SpectraMax M5 (Molecular Devices) plate reader at 20 °C. All FP data were well fitted to a 1:1 binding model and were expressed as follows:

$$\text{FP}={\text{FP}}_{\text{ini}}+\frac{\max }{2\text{nR}}\times \left({K}_{d}+\text{P}+\text{nR}-\sqrt{-4\text{nPR}+{\left({K}_{d}+\text{P}+\text{nR}\right)}^{2}}\right)$$

where FP is the observed total polarization, FP_ini_ is the initial FP of RNA without any protein, P is the protein concentration, R is the concentration of labeled RNA, n is the binding stoichiometry (protein: RNA ratio), and *K*_*d*_ is the equilibrium dissociation constant. Standard errors were obtained by fitting the data to the above equation.

### Cell culture

HEK293T cells were cultured in Dulbecco’s Modified Eagle Medium (DMEM, Gibco) supplemented with 10% (v/v) fetal bovine serum and 1% penicillin/streptomycin. Cells were grown at 37 °C in a humidified atmosphere containing 5% CO_2_.

### Plasmids and stable cell lines

All short hairpin RNAs (shRNAs) against METTL15 were inserted into PLKO.1 vectors. The modified PLKO.1 plasmid was packaged into lentivirus in HEK293T cells by PSPAX2 and PMD.2 G plasmids. HEK293T cells were infected with lentivirus followed by puromycin (1 μg/mL) selection to establish stable cell lines.

### Western blotting assay

Cells lysates were prepared in radioimmunoprecipitation assay buffer (50 mM Tris–HCl, pH 8.0, containing 150 mM NaCl, 5 mM EDTA, 0.1% SDS, and 1% NP-40) supplemented with protease inhibitor cocktails (Roche). Equal amounts of total cell lysate were separated by SDS-PAGE.

To detect histone lactylation levels, the extracted nuclear fractions from cells were lysed with nuclear buffer (50 mM Tris-HCl, pH 7.5, 500 mM NaCl, 1 mM EDTA, 0.2% NP-40, 10 mM β-mercaptoethanol, 10% glycerol) supplemented with protease inhibitor cocktail, and the nuclear lysates were sonicated using an Ultrasonic Cell Disruptor (Scientz). Protein lysate supernatant was collected after centrifugation at 15,000× *g* for 10 min at 4 °C, and was quantified with a Bradford assay kit (Sangon Biotech, C000164-0200). Equal amounts of protein were subjected to 5%–12% SDS-PAGE.

Primary antibodies against the following proteins were used in western blotting: METTL15 (Abcam, Cat# ab307819), METTL17 (Thermo, Cat# pA5-26973), hsRBFA (Thermo, Cat# pA5-59587), MT-CO1 (Abcam, Cat# ab203912), MT-CO2 (Thermo, Cat# A-6404), MT-CYB (Abcam, Cat# ab219823), MT-ND3 (Abcam, Cat# ab192306), MT-ATP8 (Proteintech, Cat# 26723-1-AP), H4K12-lac (PTM, Cat# 1411RM), H3K9-lac (PTM, Cat# 1419RM), H3K18-lac (PTM, Cat# 1406RM), H3K56-lac (PTM, Cat# 1421RM), H4K5-lac (PTM, Cat# 1407RM), H4K8-lac (PTM, Cat# 1415RM) and H4K16-lac (PTM, Cat# 1417RM). β-Tubulin (Abcam, Cat# T0023) served as a loading control. Horseradish peroxidase-conjugated anti-rabbit or anti-mouse (Bio-Rad) secondary antibodies were used to detect primary antibody binding (Respective primary antibodies), and the signal was detected using LAS4000mini (GE bio. Inc.).

### Real-time fluorescence quantitative PCR (qRT-PCR)

TRIzol (Invitrogen, Cat# 15596018) was used to extract total RNA from HEK293T cells. The isolated RNA was reverse transcribed into cDNA for qRT-PCR. SYBR Green I fluorescent dye and a Roche LC96P temperature gradient fluorescence quantitative PCR instrument were used, with GAPDH as an internal reference. All the primer sequences used in this study were listed in the Supplementary Table S2.

### Mitochondrial respiratory and glycolysis activity assay

Mitochondrial respiratory and glycolytic activities in HEK293T cells were measured by using the Seahorse Extracellular Flux Analyzer XFp (Agilent Technologies) with the XF Cell Mito Stress Test Kit (Agilent Technologies). Transfected cells (1.5 × 10^4^) were counted using ADAM-MC2 (NanoEntek) and plated into V3-PS 96-well plates the day before performing the assay. Standard mitochondria stress tests were performed by first measuring basal values followed by measurements after sequential addition of 1 µM oligomycin, 1.25 µM FCCP, and 0.5 µM rotenone/antimycin A. The glycolysis stress test was performed by first measuring basal values followed by measurements after sequential addition of 10 mM glucose, 1 µM oligomycin, and 50 mM 2-deoxy-glucose.

### ROS and membrane potential detection

MitoSOX (Yeasen, Cat# 40778ES50) was used for the determination of mitochondrial ROS. TMRE (Beyotime, Cat#C2001S) was used for the determination of membrane potential. The corresponding channels were detected by flow cytometry.

### Untargeted liquid chromatography with tandem mass spectrometry analysis

For each sample, 7 × 10^6^ HEK293T cells were added into 2 mL thickened centrifuge tubes, simultaneously, 2 magnetic beads and 10 μL of the prepared internal standard 1 were added to each sample at the same time. Then 800 µL of precooled extraction reagent (methanol: acetonitrile: water as 2:2:1 in volume) was added and ground subsequently at 50 Hz for 5 min. Following incubation at −20 °C for 2 h, samples were centrifuged at 25,000× *g* for 15 min. The supernatant was transferred into split-new EP tubes for vacuum freeze-drying. The metabolites were resuspended in 120 µL of 50% methanol and centrifuged for 15 min at 25,000× *g*, and the supernatants were transferred to autosampler vials for analysis. 10 μL of each sample was taken and mixed into QC samples to evaluate the reproducibility of the analysis. In this experiment, Waters 2777c UPLC (Waters, USA) in line with Q exactive HF high resolution mass spectrometer (Thermo Fisher Scientific, USA) was used for the separation and detection of metabolites. The mass spectrometry data was imported into Compound Discoverer 3.0 (ThermoFisher Scientific) for data processing. Metabolite identification was conducted against the BGI metabolome database (Shenzhen, China), mzCloud (ThermoFisher Scientific), ChemSpider, the Kyoto Encyclopedia of Genes and Genomes (KEGG) and the Human Metabolome Database (HMDB). The metaX software was used for further statistical analysis^52^.

### Supplementary information

Supplementary Information

Supplementary Tables S3

## Data Availability

The coordinates and structure factors for the structures of METTL15 were deposited into the Protein Data Bank under accession codes 8IPI, 8IPK, 8IPL and 8IPM.
