# Supplementary material for: Structural insights into the specific recognition of mitochondrial ribosome-binding factor hsRBFA and 12 S rRNA by methyltransferase METTL15
Source: Cell Discov. 2024 Jan 30;10:11. doi: 10.1038/s41421-023-00634-z (PMC10828496; doi:10.1038/s41421-023-00634-z)
Supplement: Supplementary file 1 — Supplementary Information [file 41421_2023_634_MOESM1_ESM.pdf]

Supplementary Information

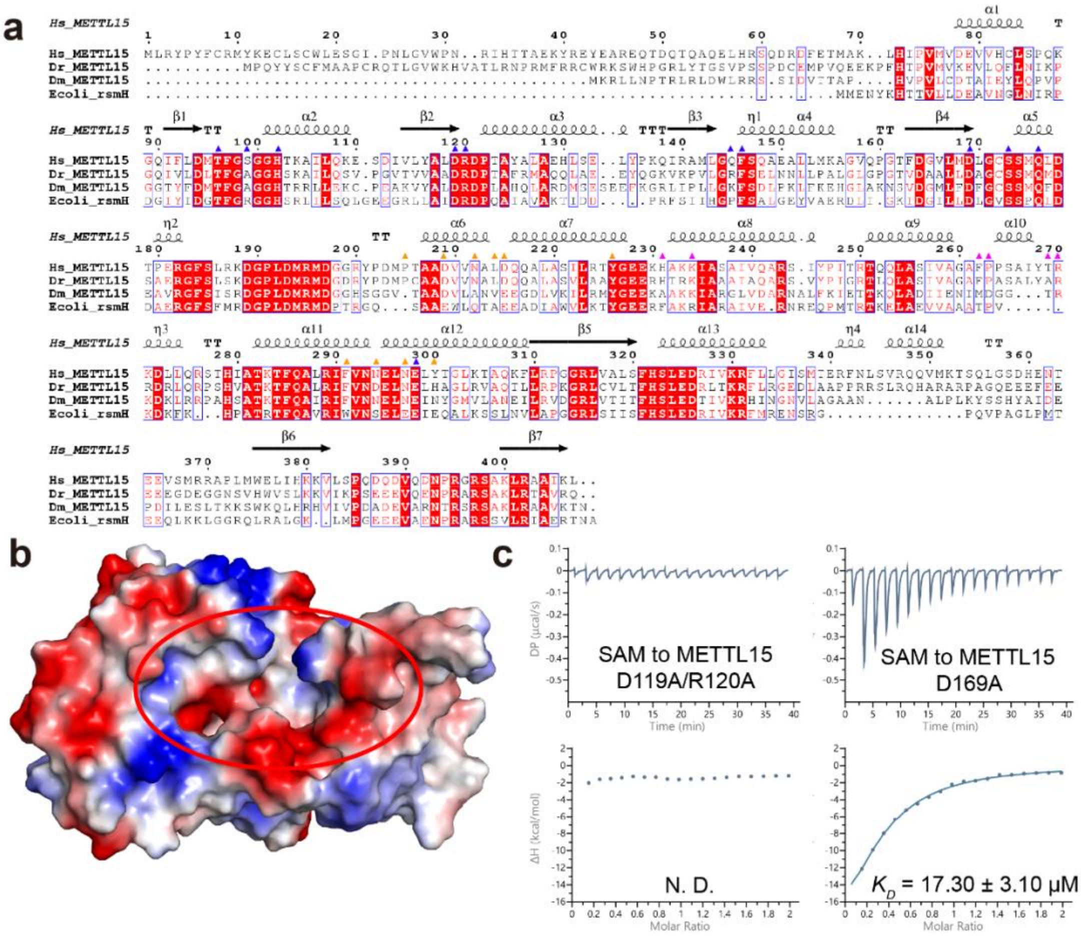

Supplementary Figure S1. (a) Sequence alignment of METTL15 from different organisms: *Homo sapiens* (*Hs*) METTL15 and the orthologs from *Drosophila melanogaster* (*Dm*), *Danio rerio* (*Dr*) and *E. coli.* homology methyltransferase RsmH. The secondary structure elements of METTL15 are shown above the sequence, as helices (for α-helices) and arrows (for β-strands). The critical residues required for the recognition of SAM, RBFA and RNA are marked as blue, bright orange and magenta triangles, respectively. (b) The electrostatic potential of the apo METTL15 is shown, in which positively charged, negatively charged and neutral areas are represented in blue, red and white, respectively. The SAM active pocket of METTL15 is highlighted as solid red circle. (c) The ITC fitting results of METTL15 mutants by SAM.

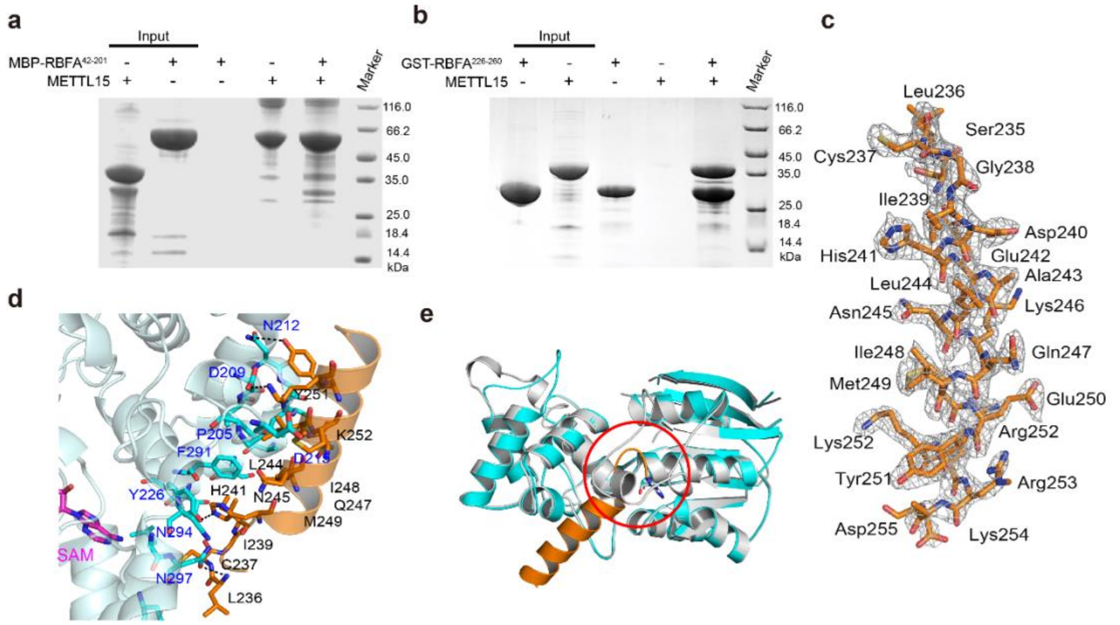

Supplementary Figure S2. (a) The pull-down assay of MBP-hsRBFA$^{42\text{-}201}$ with METTL15 was visualized by Coomassie blue staining. (b) The pull-down assay of GST-hsRBFA$^{226\text{-}260}$ with METTL15 visualized by Coomassie blue staining. (c) *2Fo-Fc* composite omit electron density map for the C-terminal helix of hsRBFA in the ternary complex (contoured at 1σ level). (d) The binding interface between METTL15 (cyan) and hsRBFA (bright orange) is shown. The critical residues required for the recognition between METTL15 (labeled blue) and hsRBFA (labeled black) are shown in stick mode. (e) The superposition of the METTL15$^{70\text{-}407}$-hsRBFA$^{226\text{-}260}$-SAM ternary complex (METTL15 is colored in cyan and hsRBFA is colored in bright orange) and the METTL15-SAM binary complex (colored in grey) shows that η-helix (Thr339~Glu361) of apo METTL15 (highlighted as solid red circle) possesses a physical barrier with the the helix of hsRBFA in ternary complex.

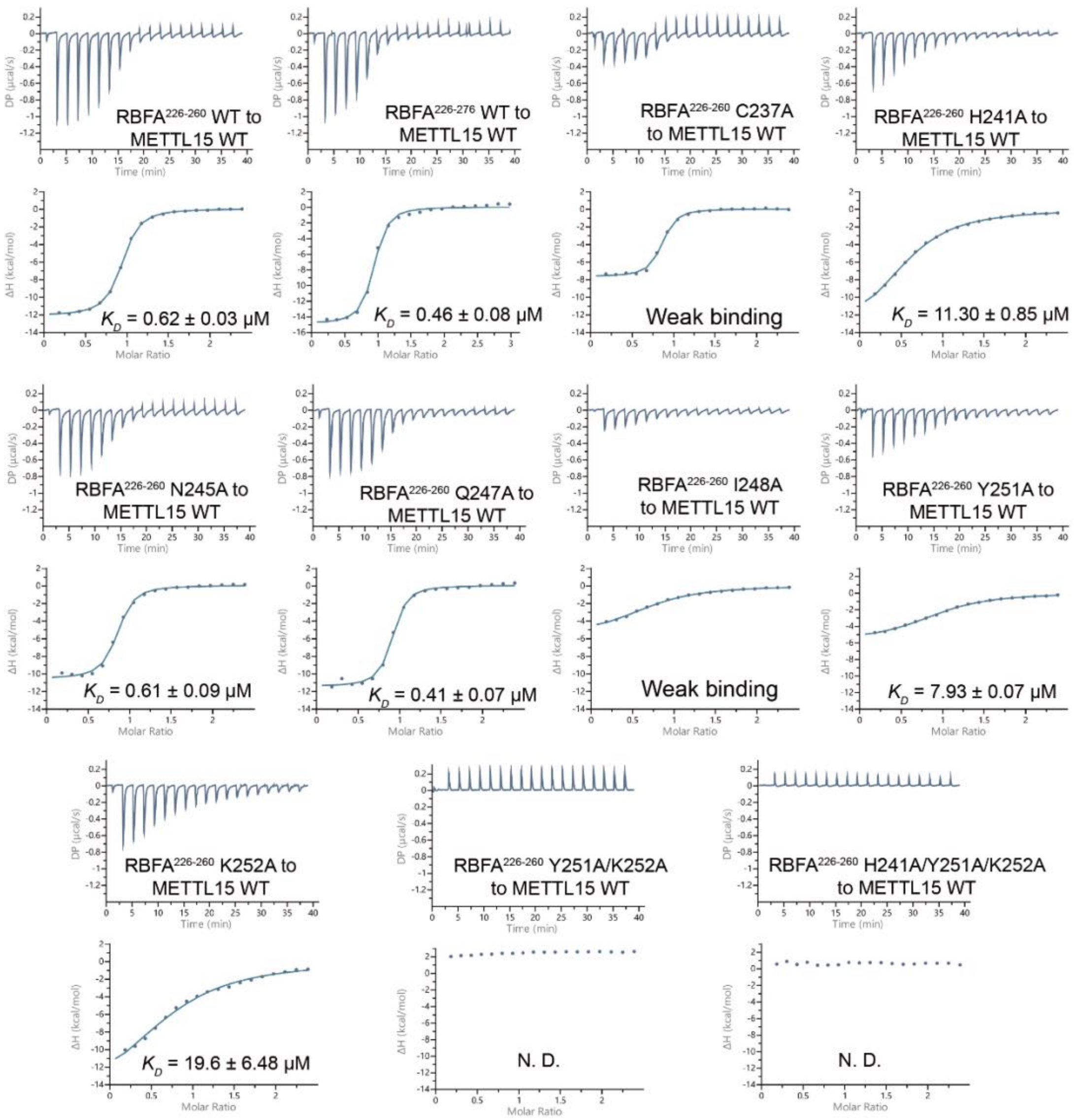

Supplementary Figure S3. The fitting curves of ITC experiments between mutated hsRBFA and wildtype METTL15 are shown.

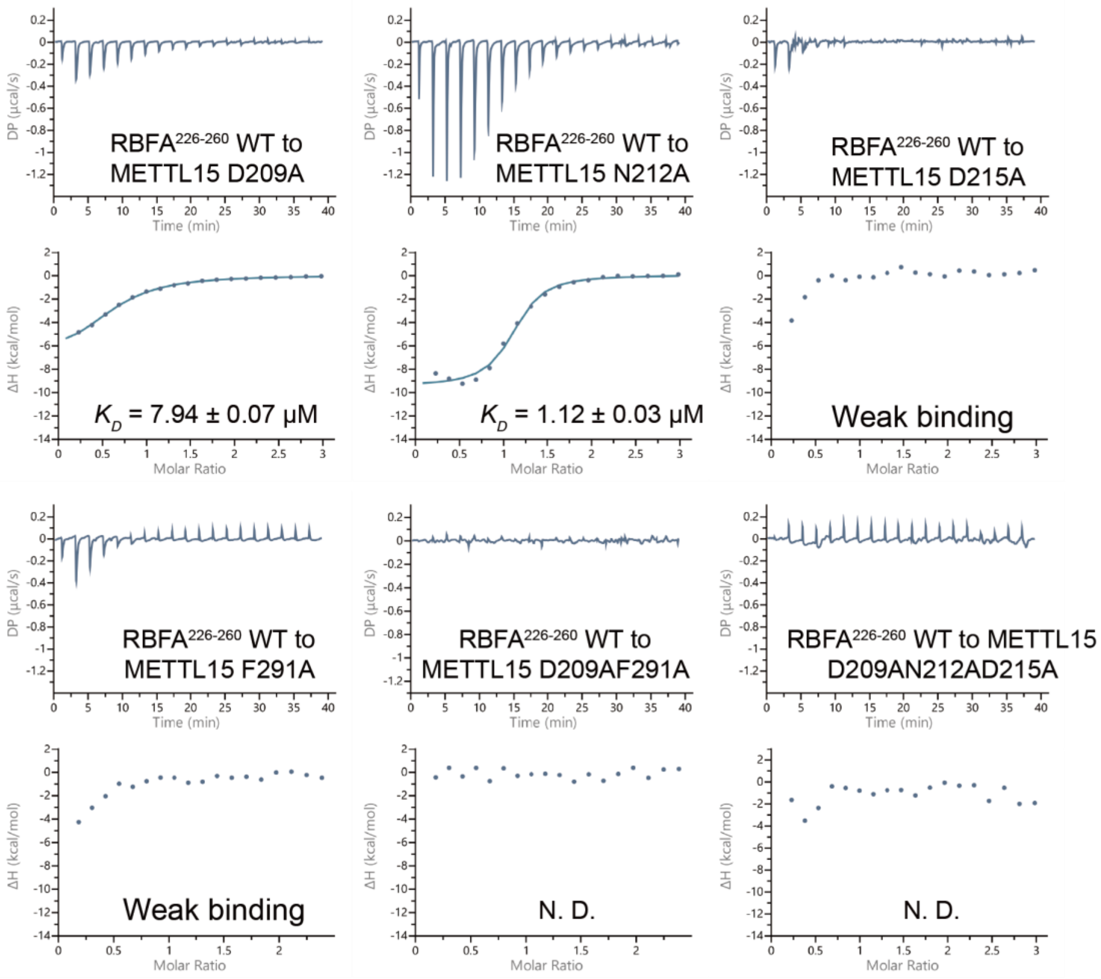

Supplementary Figure S4. The fitting curves of ITC experiments between wildtype hsRBFA and mutated METTL15 are shown.

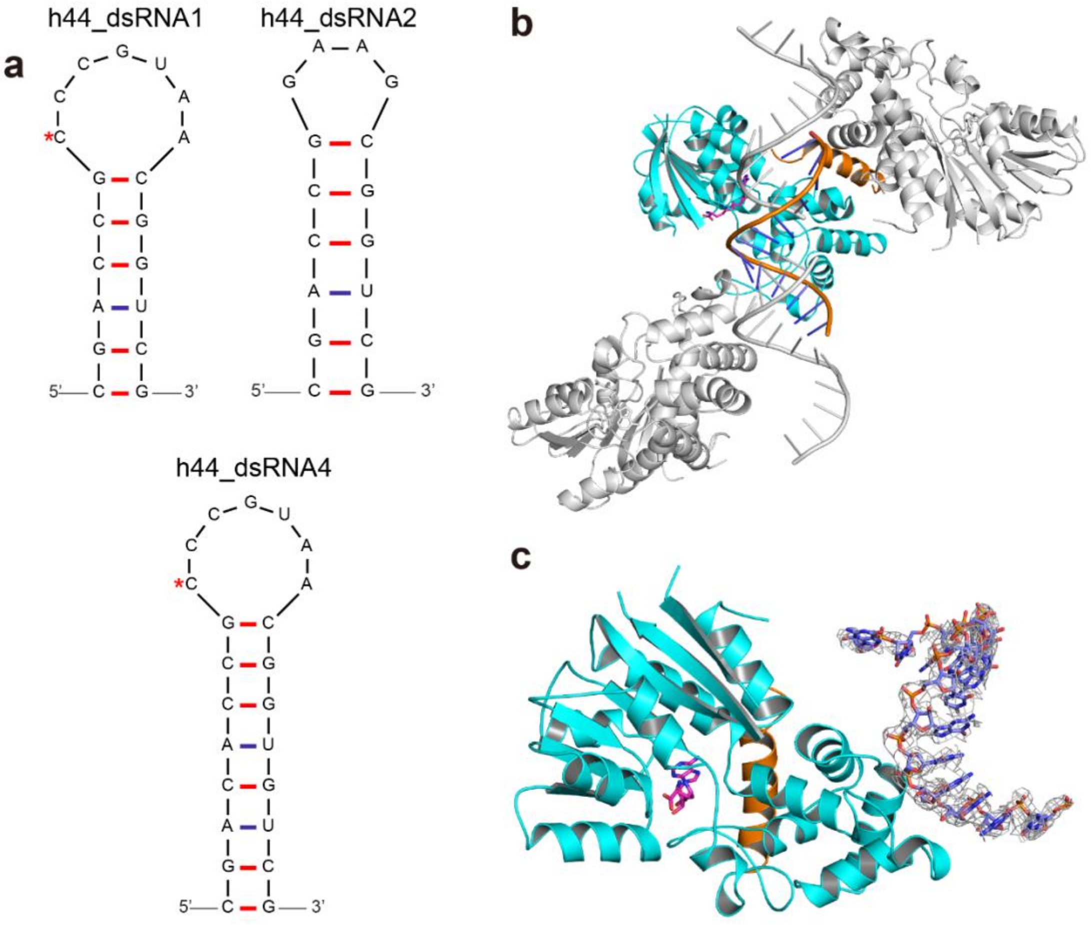

Supplementary Figure S5. (a) The predicted secondary structures of RNAs used in the experiment. The C839 residue was marked as red stat. (b) Symmetry and stereo views of the METTL15$^{70\text{-}407}$-RNA-hsRBFA$^{226\text{-}260}$-SFG quaternary complex in the crystal structure. Both the 5' end and the 3' end of h44_dsRNA1 bases in the quaternary complex partially paired with the neighboring RNA in the next symmetry equivalent. (c) *2Fo-Fc* composite omit electron density map for h44_dsRNA1 of in METTL15$^{70\text{-}407}$-RNA-hsRBFA$^{226\text{-}260}$-SFG quaternary complex (contoured at 1σ level).

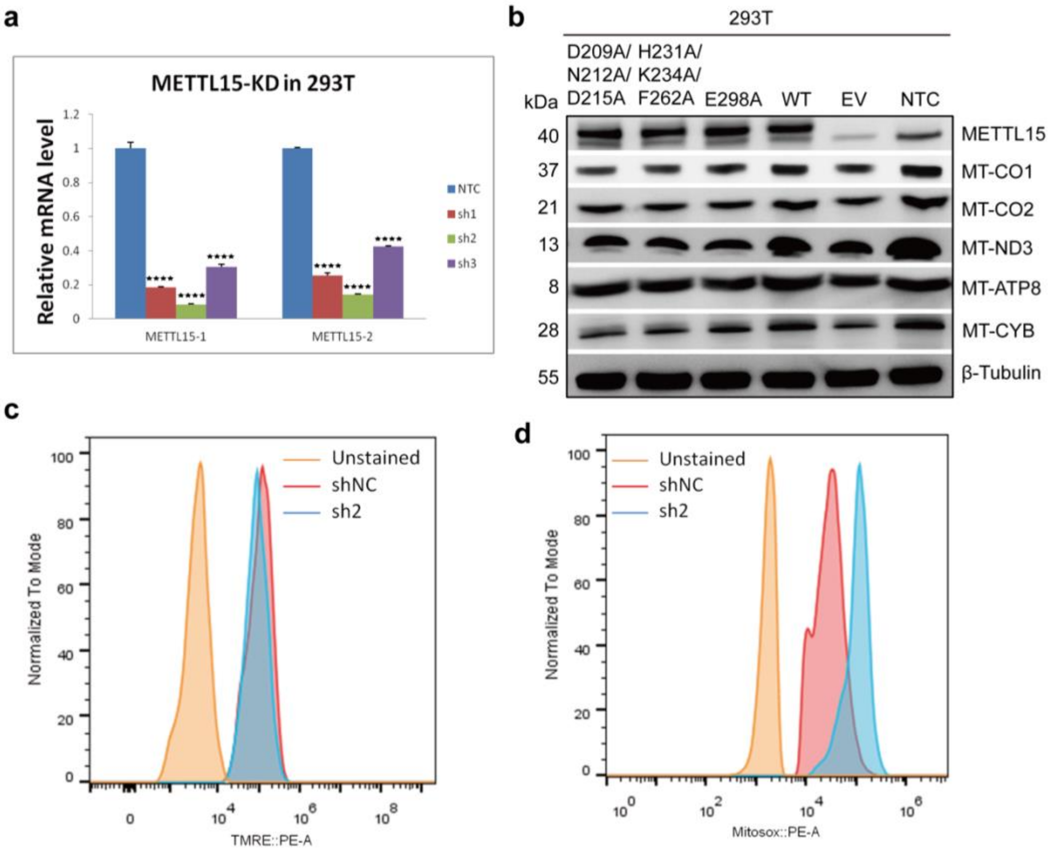

Supplementary Figure S6. (a) Quantitative real-time PCR analysis of the abundance of METTL15 mRNA transcripts in HEK293T cells treated with different shRNAs. (b) HEK293T cells in which endogenous METTL15 was knocked down using shRNA2 were further infected with viruses expressing METTL15 (wildtype, active-site mutant E298A, or the combined mutants of the interface between METTL15-hsRBFA and METTL15-RNA, D209A/N212A/D215A and H231A/K234A/F262A, respectively) followed by analysis of the mitochondrial encoded protein analysis by western blotting. β-Tubulin served as a loading control. NTC: non-targeting control. (c) Flow cytometry analysis of mitochondrial membrane potential. Unstained: cells are not stained with dye. (d) Flow cytometry analysis of cellular mitochondrial reactive oxygen species.

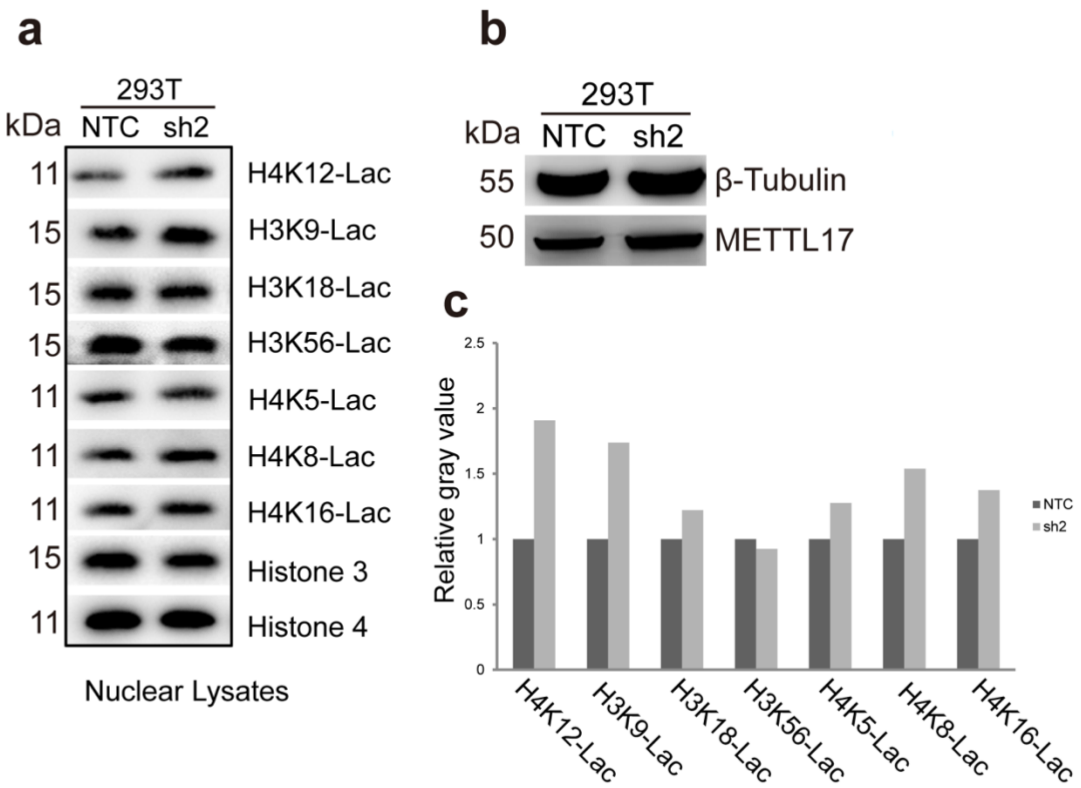

Supplementary Figure S7. (a) Western blot analyzing the levels of histone lactylation in shNC cells and METTL15 sh2 knockdown cells. NTC: non-targeting control. (b) Western blot analyzing the protein levels of METTL17 in shNC cells and METTL15 sh2 knockdown cells. β-Tubulin served as a loading control. (c) The histogram of the relative gray value in Supplementary Fig. S7a to compare the amounts of different histone lactylation modifications between sh2 knocked-down and wildtype cells. The level of each corresponding lactylation modification was normalized to histone 3 or histone 4, respectively, and set to 1 for each modification in wild-type cells.

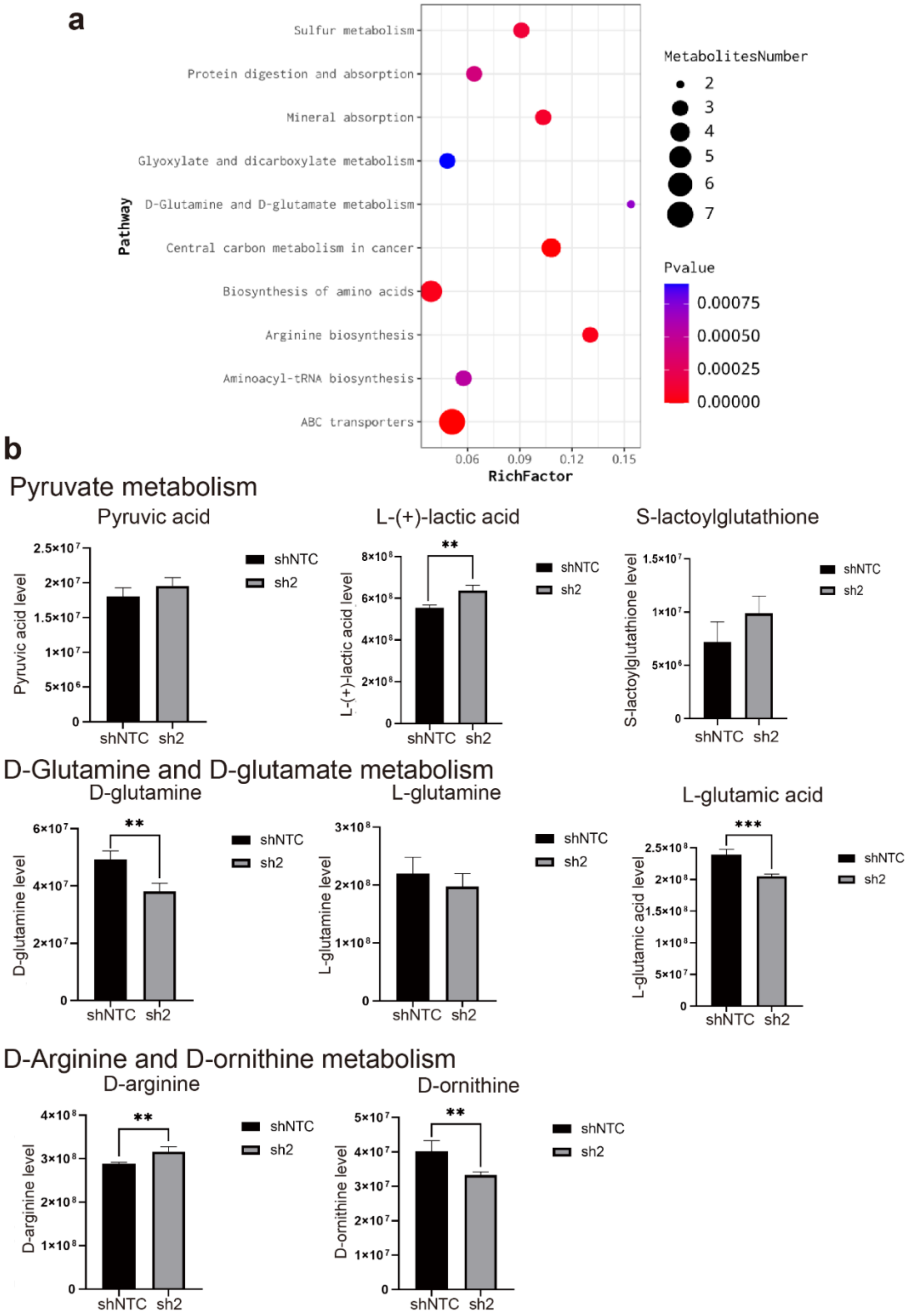

Supplementary Figure S8. (a) Metabolic pathway enrichment analysis of differential metabolites based on the KEGG database are drawn as bubble charts. (*p* value<0.05). (b) Several metabolites of the two groups of cells implicated in the pyruvate metabolism, D-Glutamine and D-glutamate metabolism, D-Arginine and D-ornithine metabolism were compared and shown as histograms.

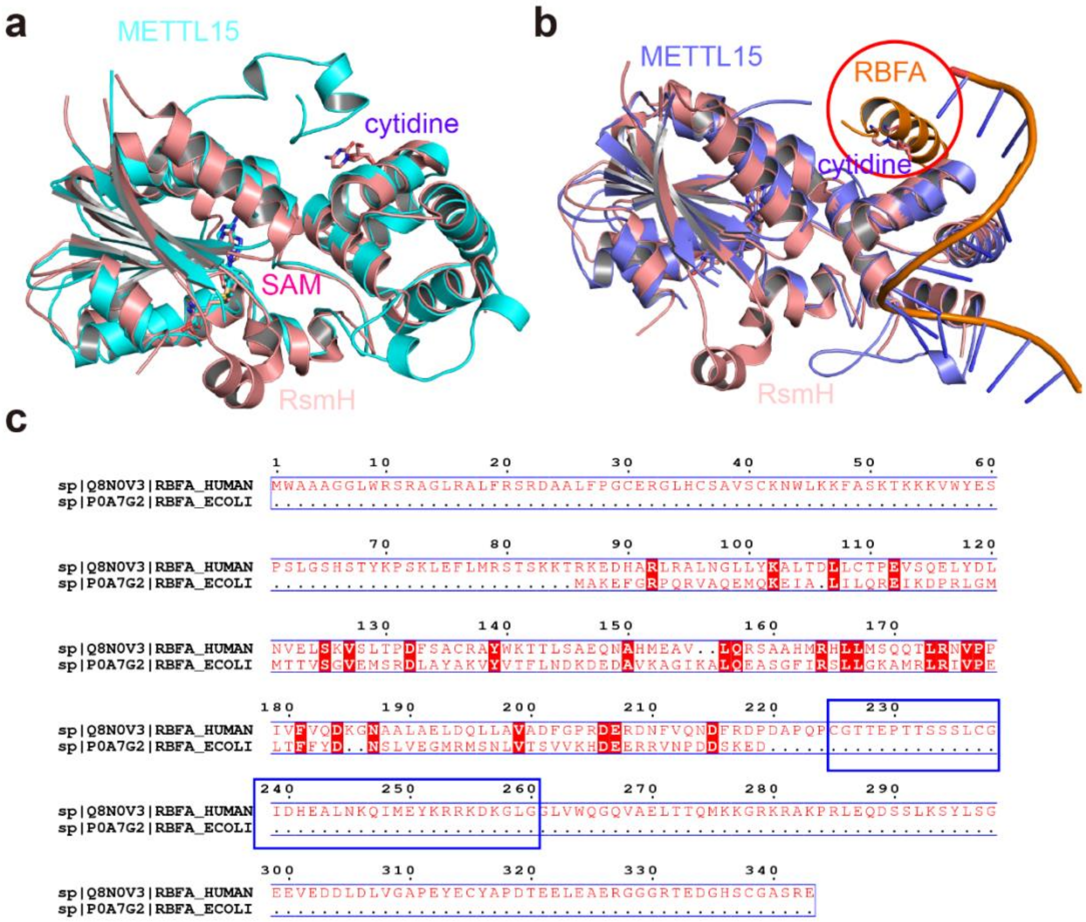

Supplementary Figure S9. (a) The superposition of the binary complex METTL15-SAM structure and RsmH structure (PDB ID 3TKA). The METTL15-SAM complex is colored in cyan and RsmH is colored in salmon. (b) The superposition of the quaternary complex METTL15$^{70\text{-}407}$-RNA-hsRBFA$^{226\text{-}260}$-SFG structure and RsmH structure. METTL15 is colored in slate, hsRBFA is colored in bright orange and RsmH is colored in salmon. The physical barrier between the helix of hsRBFA in the quaternary complex and cytidine in the RsmH structure is highlighted as a solid red circle. (c) Sequence alignment of hsRBFA and RBFA from *E coli.*. The blue square marks the C-terminal helix of hsRBFA.

Supplementary Tables

Table S1. Oligonucleotides sequence for RNA

Name	sequence (5'–3')
5’-FAM-h44_dsRNA1	5’FAM_CGACCGCCCGUAACGGUCG
h44_dsRNA1	CGACCGCCCGUAACGGUCG
5’-FAM- h44_dsRNA2	5’FAM_CGACCGGAAGCGGUCG
5’-FAM- h44_ssRNA3	5’FAM_ CACACCGCCCGUCAC
5’-FAM- h44_dsRNA4	5’FAM_CGACACCGCCCGUAACGGUGUCG
5’-FAM-polyA13	5’-FAM-AAAAAAAAAAAAA
sh1 (shRNA1)	ATGTCCCTACTTGTGAATAAT
sh2 (shRNA2)	GGCAGGCCCTTAATCCAATAT
sh3 (shRNA3)	TGGCCCTTTGGACATGAGAAT

Table S2. Primers used for qRT-PCR analysis.

Name	sequence (5'–3')
5’-hGAPDH-q	CATGTTCGTCATGGGTGTGAACCA
3’-hGAPDH-q	ATGGCATGGACTGTGGTCATGAGT
METTL15-F-1	CATGTTGGTTGGAATCTGGCA
METTL15-R-1	TGAGTTTGATCTGTTTGCTCCC
METTL15-F-2	GCCAGGAACTTTTGATGGAGT
METTL15-R-2	CCTCCCCGTATGTTCTTAGGATA
